# Socioeconomic status may affect association of vegetable intake with risk of ischemic cardio-cerebral vascular disease: a Mendelian randomization study

**DOI:** 10.3389/fnut.2023.1161175

**Published:** 2023-07-13

**Authors:** Jiutian Huang, Ziyi He, Minhui Xu, Jianing Du, Yun-tao Zhao

**Affiliations:** ^1^Aerospace Center Hospital, Peking University Aerospace School of Clinical Medicine, Beijing, China; ^2^Department of Cardiology, Aerospace Center Hospital, Beijing, China

**Keywords:** vegetable intake, socioeconomic status, ischemic cardiovascular disease, ischemic cerebrovascular disease, Mendelian randomization

## Abstract

**Background:**

Previous studies found that increasing vegetable intake benefits are reduced after adjustment for socioeconomic factors. Using genetic variation as an instrumental variable for vegetable intake and socioeconomic status, we investigated the relationship between vegetable intake and ischemic cardio-cerebral vascular diseases and focused on whether socioeconomic status was a possible confounder.

**Methods:**

From three independent genome-wide association studies, we extracted instrumental variables reflecting raw and cooked vegetable intake, which were used to perform Mendelian randomization analysis. To evaluate the effects of socioeconomic factors on vegetable intake, univariate and multivariate Mendelian randomization analyses were performed using single nucleotide polymorphisms representing education attainment and household income reported in the literature. We also performed outlier assessment and a series of sensitivity analyses to confirm the results.

**Results:**

Genetically predicted raw and cooked vegetable intake were not associated with any ischemic cardio-cerebral vascular diseases and lipid components after Bonferroni correction. Univariate Mendelian randomized analysis revealed that raw vegetable intake was positively correlated with education attainment (*β* = 0.04, *p* = 0.029) and household income (*β* = 0.07, *p* < 0.001). Multivariate Mendelian randomized model showed a positive correlation between household income and raw vegetable intake (*β* = 0.06, *p* = 0.004). Socioeconomic status was closely associated with eating habits and lifestyle related to the risk of cardiovascular diseases.

**Conclusion:**

Genetically determined raw and cooked vegetable intake was not associated with significant benefits in terms of ischemic cardio-cerebral vascular diseases while genetically determined socioeconomic status may have an impact on vegetable intake. Socioeconomic status, which was closely associated with other eating habits and lifestyle, may affect the association between vegetable intake and ischemic cardio-cerebral vascular diseases.

## Introduction

Ischemic cardio-cerebral vascular diseases are the leading cause of death and decreased quality of life worldwide (1–3). Increased vegetable intake is widely recommended in the cardiovascular disease (CVD) field (4, 5). Many observational studies support the benefits of increased vegetable intake (6, 7), including lowering serum lipids and preventing chronic disease. However, the independent effects of raw and cooked vegetables on ischemic cardiovascular disease are poorly understood, and results vary in traditional epidemiological studies (8–12).

Socioeconomic status (SES), such as education attainment and household income, has a significant impact on the occurrence of cardiovascular disease (13). A large study found that those with primary education had an increased risk of cardiovascular disease, cardiovascular event mortality, and all-cause mortality compared with those with higher education (14). Patients with financial barriers are at higher risk of future CVD events (15). Socioeconomic status can limit access to fresh vegetables and fruits due to a lack of health literacy and high prices (16, 17). Several studies found that increasing vegetable intake benefits are reduced after adjustment for socioeconomic factors (8, 10, 18). And the risk of ischemic cardio-cerebral vascular disease is related to various lifestyle factors, including diet, smoking, drinking, physical inactivity, etc. (19–22), which are also closely related to education attainment and household income (14, 23, 24).

The Mendelian randomization (MR) approach has been widely used to assess the causal effects of risk factors on disease. Relying on pooled data from genome-wide association studies (GWAS) (25), the MR approach uses genetic variants, randomly assigned to individuals at conception, as instrumental variables (IVs) to analyze causal relationships between exposure and outcome. When performed carefully, MR analysis largely overcomes the limitations of confounders and avoids the bias of typical observational studies (26). Several previous Mendelian randomization studies (12, 27, 28) have found that vegetable intake did not reduce certain metabolic risk factors and the risk of some cardiovascular events. We aimed to estimate the effects of vegetable intake on ischemic cardio-cerebral vascular disease risk and serum lipids and to focus on the influence of socioeconomic status on vegetable intake, other eating habits, and lifestyle.

## Methods

### Study design

**Figure 1:**
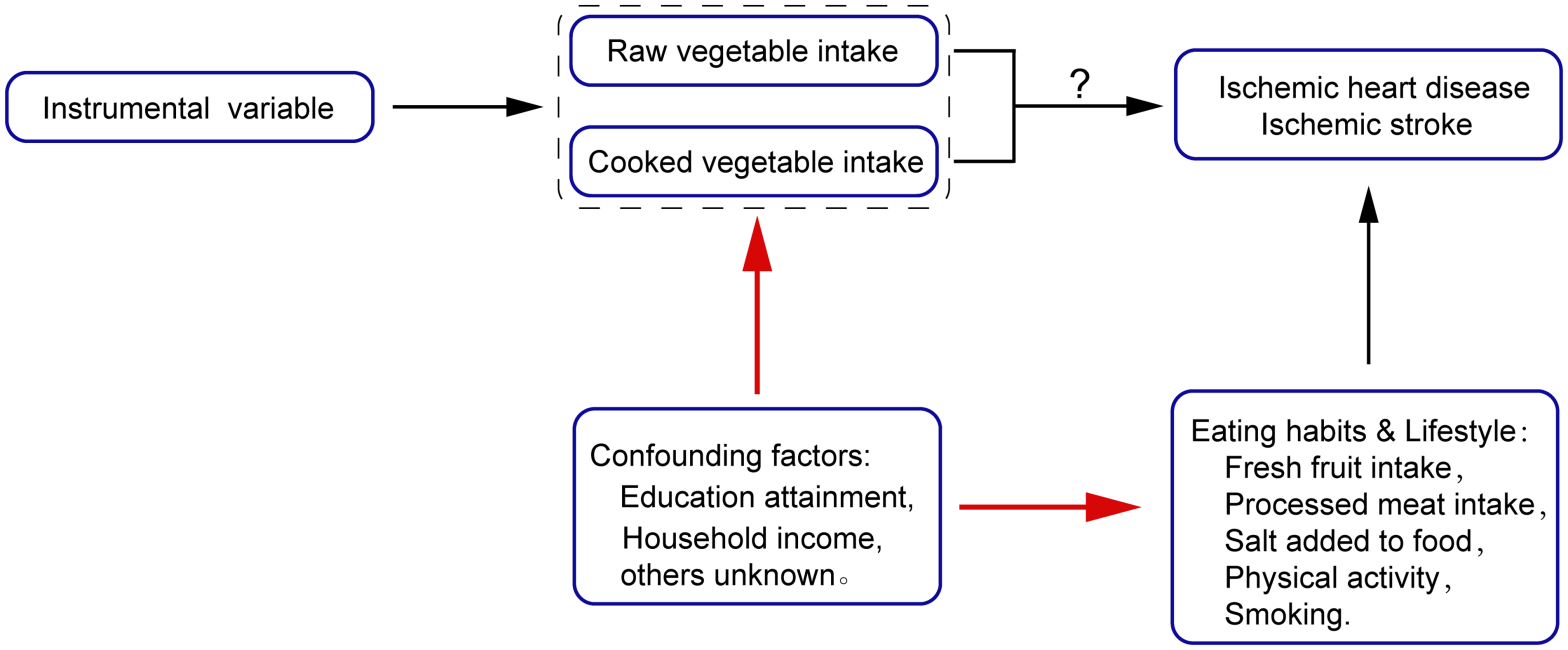
Mendelian Randomization (MR) Model of our study.

We designed a one-sample Mendelian Randomization study to evaluate how vegetable intake was related to ischemic heart disease and ischemic stroke. We selected angina, acute myocardial infarction, chronic ischemic heart disease, and cerebral infarction as our outcome variables. Considering the genetic connection between instrumental variables used for the intake of raw and cooked vegetables, both univariable and multivariable MR studies were used to assess the causal relationship between exposure and outcome. After discovering a lack of clear causal relationship between vegetable intake and ischemic cardiovascular events, we further conducted a multivariable Mendelian Randomization study to estimate the potential impact of socioeconomic status on vegetable intake, and we also attempted to prove that this impact is equally widespread in other dietary habits and lifestyle (Figure 1).

### Instrumental variable selection

Genetic instrumental variables reflecting vegetable intake were derived from three independent UK Biobank-based GWAS (Supplementary Table S1) (29–31). The measures of dietary intake, including vegetable intake, in UK Biobank are based on self-reported questionnaire data. We identified single nucleotide polymorphisms (SNPs) with a genome-wide significant *p* value (<5 × 10^−8^) associated with phenotypes. After removing duplicate SNPs, we obtained 39 SNPs reflecting raw vegetable intake, and 25 reflecting cooked vegetable intake. The SNP rs12629972 was excluded because it reflected both raw and cooked vegetable intake. We also excluded palindrome SNPs with intermediate allele frequencies (>0.42) or indel genetic variants. To account for the possibility that the three GWAS may have identified interrelated SNPs as reflecting vegetable intake, we used LDlink to remove SNPs in linkage disequilibrium (32). If a pair of SNPs had LD R^2^ > 0.01, the SNP with the higher *p* value was removed. Finally, we used PhenoScanner, a curated database of publicly available results from large-scale genetic association studies in humans (33), to check whether each SNP was associated with potential confounders (*p* < 5 × 10^−8^, proxies for European: *R*^2^ > 0.8). Rs11191193 was excluded because of its significant genetic association with educational attainment, and rs838133 because it was directly associated with total cholesterol. Finally, we used 18 SNPs reflecting raw vegetable/salad intake and 11 SNPs reflecting cooked vegetable intake for MR analysis (Supplementary Tables S2, S3). Supplementary Figures S1, S2 show the detailed selection process. Proxy SNPs (minimum linkage disequilibrium *R*^2^ = 0.8) were used for vegetable intake-associated SNPs that were unavailable in outcome datasets. Some studies have shown that these SNPs may influence people’s dietary preferences and alter genetically determined raw and cooked vegetable intake through senses such as olfaction and taste (34, 35).

In addition, in order to get a more definite conclusion, we also directly extracted the instrumental variables from MRC-IEU. The visualization results of the exposed GWAS can be obtained in Supplementary Figures S3, S4. 18 and 17 SNP were used to reflex raw and cooked vegetables, respectively. Considering that some of the SNPs may have never been reported or used, we calculated the *F* statistic ($F=\frac{N-k-1}{k}\times \frac{R^{2}}{1-R^{2}}$) for each SNP to test for a weak instrumental variable bias. F statistics for all variables were over 10, and weak instrument bias was avoided in principle (Supplementary Tables S4, S5) (36).

Genetic associations with education attainment were obtained from the GWAS conducted under the auspices of the Social Science Genetic Association Consortium, which reported 74 genome-wide significant loci associated with educational attainment in people of European descent (*n* = 293,723) (37). Educational attainment was measured by the number of years of schooling completed (EduYears, mean = 14.3, SD = 3.6). 12 loci that were unavailable in the target data-set were removed and the remaining 62 loci have been retained (Supplementary Table S6). The SNPs used as instrumental variables related to household income were derived from Shi et al. (38). The same method was used to extract the genetic association of the 54 SNPs with education attainment and vegetable intake. Of 54 SNPs, 5 were unavailable and removed; the remaining 49 SNPs were retained to perform further analysis (Supplementary Table S7). SNPs associated with education attainment may affect years of education by altering neurodevelopment at different stages. For example, rs4500960 may be related to developmental biology, brain size, and cerebra core methodology, while rs61160187 is closely related to transcription factor binding and negative regulation of signal transmission (37). And SNPs associated with household income are associated with intracranial volume, infant head circumference, and level of cognitive ability (39).

### Data sources for outcome of the Mendelian randomization analysis

The primary outcomes included ischemic cardio-cerebral vascular diseases diagnosed by the International Classification of Diseases version, Tenth Version, with the following ICD-10 codes: I20 (angina pectoris), I21 (acute myocardial infarction), I25 (chronic ischemic heart disease), and I63 (cerebral infarction). Data were acquired from the UK Biobank summary statistics curated in the MRC-IEU Open GWAS database or provided by Neale Lab (40). Serum lipid and lipoprotein levels were measured using the Nightingale high-throughput NMR metabolomics platform in 2020, and blood samples were provided by UK Biobank. GWAS results based on these metabolic biomarkers can be obtained from MRC-IEU (European, *N* = 115,078).

The raw and cooked vegetable intake as well as vegetarian alternatives intake used as outcome variables in MVMR were obtained from MRC-IEU. To illustrate the broad impact of education attainment and household income on IHD risk was not limited to the preferences for vegetable intake, we also obtained a series of outcome variables related to diet and lifestyle, including fresh fruit intake, processed meat intake, salt add to food and smoking (from MRC-IEU) and physical activity (from Within family GWAS consortium). Source and relevant information of result variables used in the study can be acquired from Supplementary Table S8.

### Statistical analysis

We used the inverse-variance weighted (IVW) method as the main method to evaluate the causal effect of exposure and outcome. Due to the potential genetic association between raw and cooked vegetables, as well as between education and household income, we used the multivariate Mendelian method to analyze their independent effects. The SNPs used to conduct multivariable MR were combinations of instrumental variables of each exposure in univariable MR. We calculated the odds ratio (OR) and 95% confidence interval (CI). Cochrane’s Q-statistic was used to assess the heterogeneity of SNP effects (Supplementary Tables S15, S16). If significant heterogeneity was observed, a random-effects IVW model was applied. For sensitivity analyses, we used three complementary methods with different assumptions for valid estimates. MR-Egger lacks statistical power for assessment of causal effects and provides wider confidence intervals, but can detect horizontal pleiotropy by *p* value for its intercept term (Supplementary Table S14) (41). A weighted median method generates homogeneous causal estimates, although >50% of weights derived in the analysis arise from invalid instrumental variables (42). Mendelian Randomization Pleiotropy RESidual Sum and Outlier (MR-PRESSO) can identify outlying SNPs and correct for horizontal pleiotropy through their exclusion (Supplementary Table S13) (43). Additionally, we performed leave-one-out sensitivity analysis to identify potentially highly influential SNPs (Supplementary Figures S5–S12).

All statistical analyses were performed in R (version 4.1.3, R Foundation for Statistical Computing, Vienna, Austria), with MR analyses performed using the TwoSampleMR package (version 0.5.6) (44), MRPRESSO package (version 1.0) (43), and MVMR package (45). Statistical significance was set at a two-sided *p* value < 0.05. Bonferroni correction of the evidential threshold was based on the number of exposures (*p* value <0.05/the number of exposures).

## Results

### Association of genetically predicted vegetable intake with ischemic cardio-cerebral vascular disease and lipid profile

Genetically predicted raw and cooked vegetable intake (IVs from three UK biobank based GWAS) were not associated with any ischemic cardiovascular diseases according to IVW analysis (P>0.05) (Table 1). The multivariate MR (IVs from three UK biobank based GWAS) observed a correlation between cooked vegetable intake and an increased risk of Chronic ischemic heart disease (OR 1.02; 95% CI 1.00, 1.04; *p* = 0.042) (Table 1), but did not exceed the significance level of Bonferroni correction (*p* > 0.0125). IVW analysis also showed that genetically predicted raw and cooked vegetable intake (IVs from MRC-IEU) were not associated with any ischemic cardiovascular diseases (Supplementary Table S9; Supplementary Figures S13, S14).

**Table 1:** Estimates given as odds ratios (ORs) and 95% confidence intervals for the effect of raw and cooked vegetable intake on ischemic cardio-cerebral vascular diseases.

Outcomes	Method	Raw vegetable intake				Cooked vegetable intake			
		SNPs	OR	95% CI	*p*-value	SNPs	OR	95% CI	*P*-value
Angina pectoris	MR-Egger	18	1.01	0.98 ~ 1.05	0.405	11	0.97	0.91 ~ 1.02	0.243
	Weighted median	18	1.00	0.99 ~ 1.02	0.782	11	1.01	0.99 ~ 1.02	0.587
	Inverse variance weighted	18	1.01	0.99 ~ 1.02	0.421	11	1.00	0.98 ~ 1.01	0.692
	MR-PRESSO(Outlier-corrected)	NA	NA	NA	NA	NA	NA	NA	NA
	MVMR	18	1.00	0.99 ~ 1.01	0.915	11	1.00	0.98 ~ 1.01	0.659
Myocardial infarction	MR-Egger	18	1.01	0.99 ~ 1.04	0.341	11	1.02	0.96 ~ 1.08	0.51
	Weighted median	18	0.99	0.98 ~ 1.01	0.372	11	1.02	1.00 ~ 1.04	0.038
	Inverse variance weighted	18	1.00	0.99 ~ 1.01	0.909	11	1.01	1.00 ~ 1.02	0.18
	MR-PRESSO(Outlier-corrected)	NA	NA	NA	NA	11	1.01	1.00 ~ 1.03	0.05
	MVMR	18	0.99	0.98 ~ 1.00	0.202	11	1.01	1.00 ~ 1.03	0.056
Chronic ischaemic heart disease	MR-Egger	18	1.04	1.00 ~ 1.08	0.067	11	1.04	0.95 ~ 1.14	0.443
	Weighted median	18	1.00	0.98 ~ 1.02	0.786	11	1.02	0.99 ~ 1.04	0.229
	Inverse variance weighted	18	0.99	0.98 ~ 1.01	0.424	11	1.02	0.99 ~ 1.04	0.156
	MR-PRESSO(Outlier-corrected)	NA	NA	NA	NA	NA	NA	NA	NA
	MVMR	18	0.98	0.97 ~ 1.00	0.075	11	1.02	1.00 ~ 1.04	0.042
Cerebral infarction	MR-Egger	18	1.01	0.99 ~ 1.02	0.573	11	0.99	0.95 ~ 1.03	0.582
	Weighted median	18	1.00	0.99 ~ 1.01	0.701	11	1.00	0.99 ~ 1.01	0.933
	Inverse variance weighted	18	1.00	0.99 ~ 1.01	0.417	11	1.00	0.99 ~ 1.01	0.561
	MR-PRESSO(Outlier-corrected)	NA	NA	NA	NA	NA	NA	NA	NA
	MVMR	18	1.00	0.99 ~ 1.01	0.874	11	0.99	0.98 ~ 1.00	0.285

IVW analysis showed that genetically predicted raw vegetable intake (IVs from three UK biobank based GWAS) was associated with reduced low-density lipoprotein cholesterol (LDL-C) (*β* −0.25; 95% CI −0.44, −0.07; *p* = 0.006) and apolipoprotein B (ApoB) (*β* −0.28; 95% CI −0.43, −0.12; *p* < 0.001) (Supplementary Tables S10, S11). MVMR analysis showed that raw vegetable intake was only associated with reduced ApoB (*β* −0.22; 95% CI −0.38, −0.06; *p* = 0.013), but did not exceed the significance level of Bonferroni correction (*p* > 0.0083) (Supplementary Tables S10, S11). Cooked vegetable intake did not significantly influence serum lipid components according to the IVW and MVMR analysis. Results of Mendelian randomization analysis of genetically predicted vegetable intake (IVs from MRC-EU) and lipid profiles are presented in Supplementary Tables S12; Supplementary Figures S15, S16.

### Impact of social-economic conditions on vegetable intake

**Figure 2:**
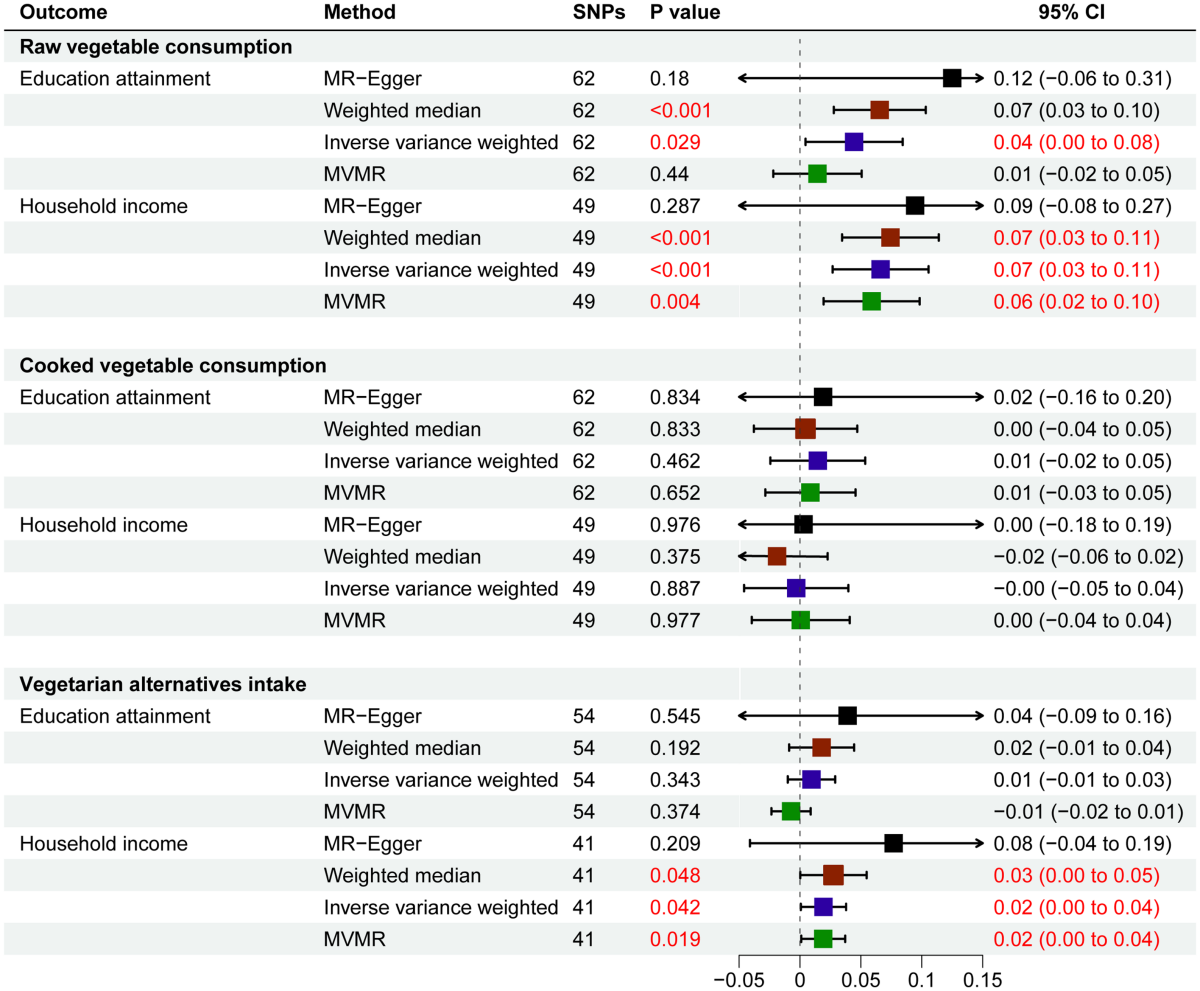
Estimates Given as Beta and 95% Confidence Intervals for the Effect of Education Attainment and Household Income on Vegetable Consumption.

We conducted univariable MR analysis to explore the impact of education attainment and household income on raw and cooked vegetable intake. People with higher education attainment tended to eat more raw vegetables (IVW: *β* 0.04; 95% CI 0.00, 0.08; *p* = 0.029). Similarly, people with higher household income tended to eat more raw vegetables (IVW: *β* 0.07; 95% CI 0.03, 0.11; P<0.001) and vegetarian alternatives intake (IVW: *β* 0.02; 95% CI 0.00, 0.04; *p* = 0.042), while we did not find the impact of social-economic conditions on cooked vegetable intake (Figure 2).

Considering that education and income are related genetically and socially, we next performed MVMR. In the MVMR model, education attainment had a weakened influence on raw vegetable intake (*β* 0.01; 95% CI −0.02, 0.05; *p* = 0.440). Household income was still positively related to raw vegetable intake (*β* 0.06; 95% CI 0.02, 0.10; *p* = 0.004) and vegetarian alternatives intake (IVW: *β* 0.02; 95% CI 0.00, 0.04; *p* = 0.019) (Figure 2).

### Impact of social-economic conditions on other eating habits and lifestyle

**Figure 3:**
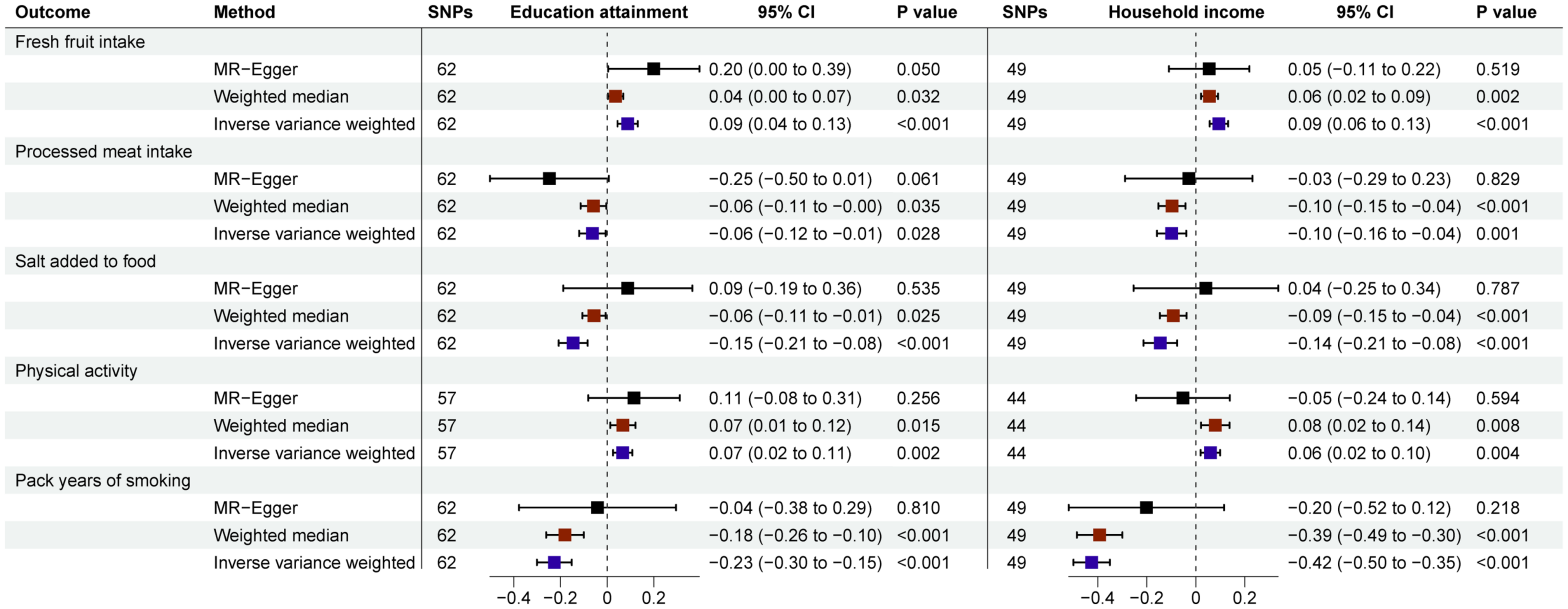
Estimates Given as Beta and 95% Confidence Intervals for the Effect of Education Attainment and Household Income on Eating Habits and Lifestyle.

Finally, we conducted univariable MR analysis to explore the impact of education attainment and household income on other eating habits and lifestyle to show that socioeconomic status has an extremely broad impact on ischemic cardio-cerebral vascular diseases. People with higher education attainment and household income tended to eat more fresh fruit intake (IVW: *β* 0.09; *p* < 0.001) (IVW: *β* 0.09; *p* < 0.001), eat less processed meat (IVW: *β* −0.06; *p* = 0.028) (IVW: *β* −0.10; *p* = 0.001), add less salt to food (IVW: *β* −0.15; *p* < 0.001) (IVW: *β* −0.14; *p* < 0.001), have more physical activity (IVW: *β* 0.07; *p* = 0.002) (IVW: *β* 0.06; *p* = 0.004) and smoke less (IVW: *β* −0.23; *p* < 0.001) (IVW: *β* −0.42; *p* < 0.001; Figure 3).

## Discussion

Our results showed that genetic variant-determined increases in raw or cooked vegetable intake did not confer a benefit in terms of ischemic cardio-cerebral vascular diseases. Increased raw vegetable intake may reduce LDL-C and ApoB levels. In addition, socioeconomic status, including income and education, have positive effects on raw vegetable intake in persons of European ancestry. Meanwhile, socioeconomic status had an extremely broad impact on other eating habits and lifestyle.

Worldwide dietary guidelines (4, 5) include recommendations for increased vegetable intake to reduce the risk of CVD, but the independent effects of raw versus cooked vegetables by different epidemiological studies have yielded inconsistent results. Leenders et al. (9) reported that vegetable intake was associated with lower CVD mortality, with a stronger association for raw vegetables. Miller et al. (10) showed that CVD incidence was only associated with high cooked vegetable intake. In a large prospective cohort study (8), total and raw vegetable intake was inversely associated with cardiovascular disease outcomes. Systematic reviews have shown that total vegetable intake is associated with reduced CVD incidence and stroke risk (46–48). A Mendelian randomization study (27) has found no evidence of an association between cooked and raw vegetable intake and coronary heart disease, heart failure, or atrial fibrillation. Since the Mendelian randomization method can reduce the unknown confounders, different results between conventional epidemiological studies and Mendelian randomization studies may be related to differences in study populations and research methods, and insufficient adjustment for confounding factors in conventional epidemiological studies. High-vegetable diets are generally lower in calories, fat, sodium, and glycemic load, which are well-established risk factors for ischemic cardio-cerebral vascular diseases (11, 49). It may be a lack of significance in merely increasing vegetable intake without controlling for other variables such as salt intake and total energy intake. And the impact of diet on health outcomes is complex. It is important to control for consistency in overall dietary patterns when assessing the health effects of single foods or nutrients (30).

LDL-C and other apolipoproteins play central roles in ischemic cardio-cerebral vascular disease occurrence and progression (50–52). Increased vegetable intake may improve plasma lipid profiles, potentially protecting against ischemic cardio-cerebral vascular diseases. Our findings indicated that increased vegetable intake did not significantly improve lipoproteins, except that raw vegetable intake may be associated with improved LDL-C and ApoB. Although increased vegetable intake has been associated with lower plasma LDL-C in observational studies (53, 54), randomized controlled trial results suggest that increased vegetable intake has negligible effects on plasma cholesterol component concentrations (55, 56). This suggests that although increased vegetable intake may have some beneficial influence on lipid profile, the benefits may be insufficient to yield clinical effects. There are several possible reasons for the different effects of raw and cooked vegetables on lipoproteins. Cooking vegetables may increase salt and fat intake, which are linked to CVD morbidity and mortality (57, 58). Cooking improves food safety and food digestibility but also impairs food quality, resulting in the loss of certain nutrients (59–61). Moreover, different types of vegetables may be eaten differently (8).

Low socioeconomic status has been associated with the development of ischemic cardio cerebral vascular disease and may confer comparable cardiovascular risks to traditional risk factors (62, 63). A prospective cohort study demonstrated that adults with low SES are at higher risk of CVD mortality and cardiovascular events than adults with high SES, partly mediated by lifestyle (64). The increased CVD burden in low SES populations may be due to a range of biological, behavioral, and psychosocial risk factors (13). In our screening of instrumental variables of vegetable intake, rs11191193 had significant genetic associations with educational attainment. Roos et al. (65) showed that household education level is an important determinant of raw vegetable intake. A large study found that individuals with primary school education had an increased risk of cardiovascular disease and cardiovascular mortality compared with those with higher education (14). An MR study (66) reported an inverse association between genetically determined educational attainment and CAD risk. And there is a strong association between genetically determined educational attainment and risk factors such as smoking, body mass index, and hypertension (66). One analysis suggested that most CHD risk among individuals with low education is due to behavioral and biological risk factors, with the main contributors being smoking, physical inactivity, and hypertension (23). The level of education may affect the ability to develop health literacy and access to healthy lifestyle and dietary recommendations (16, 67), leading to changes in vegetable intake as well as other lifestyle. Income levels have been consistently associated with CVD risk (14). Population analysis studies of atherosclerosis risk in communities have shown that living in deprived areas is associated with a higher incidence of coronary heart disease (68). The increased risk of CVD in low-income groups may be related to poor dietary choices and increased costs of healthy foods. In low-income areas with limited access, there are more fast food restaurants, fewer supermarkets, and fewer brand options, resulting in limited access to fresh fruits and vegetables (17, 69–71). Economic differences can affect not only the availability of resources but also the promotion or maintenance of a healthy lifestyle (72, 73).

Higher income and higher education attainment may be associated with improved health perceptions and lifestyle factors (74, 75), potentially explaining the positive conclusions of studies that did not fully adjust for socioeconomic status. In a large prospective cohort study (8), total and raw vegetable intake was inversely associated with cardiovascular disease outcomes. However, these associations significantly decreased after adjustment for potential confounders, such as socioeconomic status and lifestyle factors. Results from a cohort study (10) showed that higher vegetable intake was inversely associated with major cardiovascular disease, myocardial infarction, and cardiovascular mortality in models adjusted only for age, sex, and center. After multivariable adjustment including socioeconomic status, the association was significantly attenuated, and only non-cardiovascular mortality and total mortality remained significant. Similarly, in another study from China (18), higher vegetable intake was associated with lower CVD mortality risk in a minimally adjusted model, and the association was no longer significant after adjustment for factors such as socioeconomic status. Given the complex interrelationships between socioeconomic status, eating habits, lifestyle, and health outcomes, it is important to adjust for socioeconomic status to reduce bias.

### Strength

Our study has several strengths. We investigated the independent effects of raw and cooked vegetable intake on ischemic cardio-cerebral vascular diseases and lipid profiles. Compared with traditional observational studies, MR studies reduce the effects of unknown confounders and reverse causality (30). Especially, by using the Mendelian randomization analysis, we examined the impact of socioeconomic factors on vegetable intake, other eating habits, and lifestyle, confirming that the effects of education and income on dietary habits and lifestyle are relatively common and may be extremely important confounders in previous studies.

### Limitations

Our work had several limitations. Our selected GWAS for vegetable intake did not report specific intake characteristics, such as vegetable type. Therefore, we could not perform a stratified MR analysis by vegetable type, which would help draw more valuable causal inferences. Additionally, our MR study was restricted to individuals of European ancestry (76). Due to differences in genetic background and eating habits, similar studies should be conducted on a larger scale around the world. Moreover, the SNPs of the utilized GWAS were all self-reported, which may differ from the actual intake, potentially introducing some bias to the results. Individuals tend to report more healthy foods and less unhealthy foods, and people with a high BMI tend to report less food intake (77, 78). Diet changes over time may also be a potential limitation (79). Furthermore, we selected two sample datasets with a large proportion of the population overlapping, which may increase the bias in the direction of the observed association and the inflation of the Type I error rate and false-positive results (80). Finally, MR relies on assumptions that genetic tools are associated with exposure, independent of potential confounders and that genetic tools are associated with outcomes only through exposure. Although we speculated about the possible mechanism of action of the instrumental variable SNPs, the effect of the SNP on the function of the gene product is unknown and is based only on a statistical association between gene and apparent effect (81). We cannot completely avoid horizontal pleiotropic effects because it is difficult to determine the biological function of exact genetic variants. There remains a need for further high-quality GWAS and MR analyses.

## Conclusion

Our results showed that genetically determined raw and cooked vegetable intake was not associated with significant benefits related to ischemic cardio-cerebral vascular disease. Genetically determined socioeconomic status may have an impact on vegetable intake. Additionally, socioeconomic status, which was closely associated with other eating habits and lifestyle, may affect the association between vegetable intake and ischemic cardio-cerebral vascular diseases.

## Data availability statement

The original contributions presented in the study are included in the article/Supplementary material, further inquiries can be directed to the corresponding author.

## Author contributions

JH and ZH: design the study and draft the work. MX and JD: data collection and statistical analysis. Y-tZ: design the study and revise it critically for important intellectual content. All authors contributed to the article and approved the submitted version.

## Conflict of interest

The authors declare that the research was conducted in the absence of any commercial or financial relationships that could be construed as a potential conflict of interest.

## Publisher’s note

All claims expressed in this article are solely those of the authors and do not necessarily represent those of their affiliated organizations, or those of the publisher, the editors and the reviewers. Any product that may be evaluated in this article, or claim that may be made by its manufacturer, is not guaranteed or endorsed by the publisher.

## Abbreviations

ApoB: apolipoprotein B
CI: confidence interval
CVD: cardiovascular disease
IEU: Integrative Epidemiology Unit
IVs: instrumental variables
IVW: inverse-variance weighted
GWAS: genome-wide association studies
LDL-C: low-density lipoprotein cholesterol
MR: Mendelian randomization
MVMR: multivariable Mendelian randomization
OR: odds ratio
SES: socioeconomic status
SNPs: single nucleotide polymorphisms
