# Supplementary material for: Socioeconomic status may affect association of vegetable intake with risk of ischemic cardio-cerebral vascular disease: a Mendelian randomization study
Source: Front Nutr. 2023 Jul 13;10:1161175. doi: 10.3389/fnut.2023.1161175 (PMC10436213; doi:10.3389/fnut.2023.1161175)

| **List of Supplementary Figures** | |
| --- | --- |
| Figure S1 | Flowchart detailing the selection of instrument variables for raw vegetable intake |
| Figure S2 | Flowchart detailing the selection of instrument variables for cooked vegetable intake |
| Figure S3a | Manhattan plot of GWAS used for extracting instrument variables of raw vegetable intake in MRC-IEU(GWAS ID:ukb-b-1996) |
| Figure S3b | QQ plot of GWAS used for extracting instrument variables of raw vegetable intake in MRC-IEU(GWAS ID:ukb-b-1996) |
| Figure S4a | Manhattan plot of GWAS used for extracting instrument variables of cooked vegetable intake in MRC-IEU(GWAS ID:ukb-b-8089) |
| Figure S4b | QQ plot of GWAS used for extracting instrument variables of cooked vegetable intake in MRC-IEU(GWAS ID:ukb-b-8089) |
| Figure S5 | Leave-one-out sensitivity analyses for raw vegetable intake(IV from UK biobank) on ischemic cardio-cerebral vascular diseases |
| Figure S6 | Leave-one-out sensitivity analyses for cooked vegetable intake (IV from UK biobank)on ischemic cardio-cerebral vascular diseases |
| Figure S7 | Leave-one-out sensitivity analyses for raw vegetable intake (IV from UK biobank) on lipid profile |
| Figure S8 | Leave-one-out sensitivity analyses for cooked vegetable intake (IV from UK biobank) on lipid profile |
| Figure S9 | Leave-one-out sensitivity analyses for raw vegetable intake (IV from MRC-IEU) on ischemic cardio-cerebral vascular diseases |
| Figure S10 | Leave-one-out sensitivity analyses for cooked vegetable intake (IV from MRC-IEU) on ischemic cardio-cerebral vascular diseases |
| Figure S11 | Leave-one-out sensitivity analyses for raw vegetable intake (IV from MRC-IEU) on lipid profile |
| Figure S12 | Leave-one-out sensitivity analyses for cooked vegetable intake (IV from MRC-IEU) on lipid profile |
| Figure S13 | Estimates given as odds ratios (ORs) and 95% confidence intervals for the effect of raw vegetable intake (IV from UK biobank and MRC-IEU) on ischemic cardio-cerebral vascular diseases |
| Figure S14 | Estimates given as odds ratios (ORs) and 95% confidence intervals for the effect of cooked vegetable intake (IV from UK biobank and MRC-IEU) on ischemic cardio-cerebral vascular diseases |
| Figure S15 | Estimates given as beta and 95% confidence intervals for the effect of raw vegetable intake (IV from UK biobank and MRC-IEU) on lipid profiles |
| Figure S16 | Estimates given as beta and 95% confidence intervals for the effect of cooked vegetable intake (IV from UK biobank and MRC-IEU) on lipid profiles |

**Figure S1. Flowchart detailing the selection of instrument variables for raw vegetable intake**

**
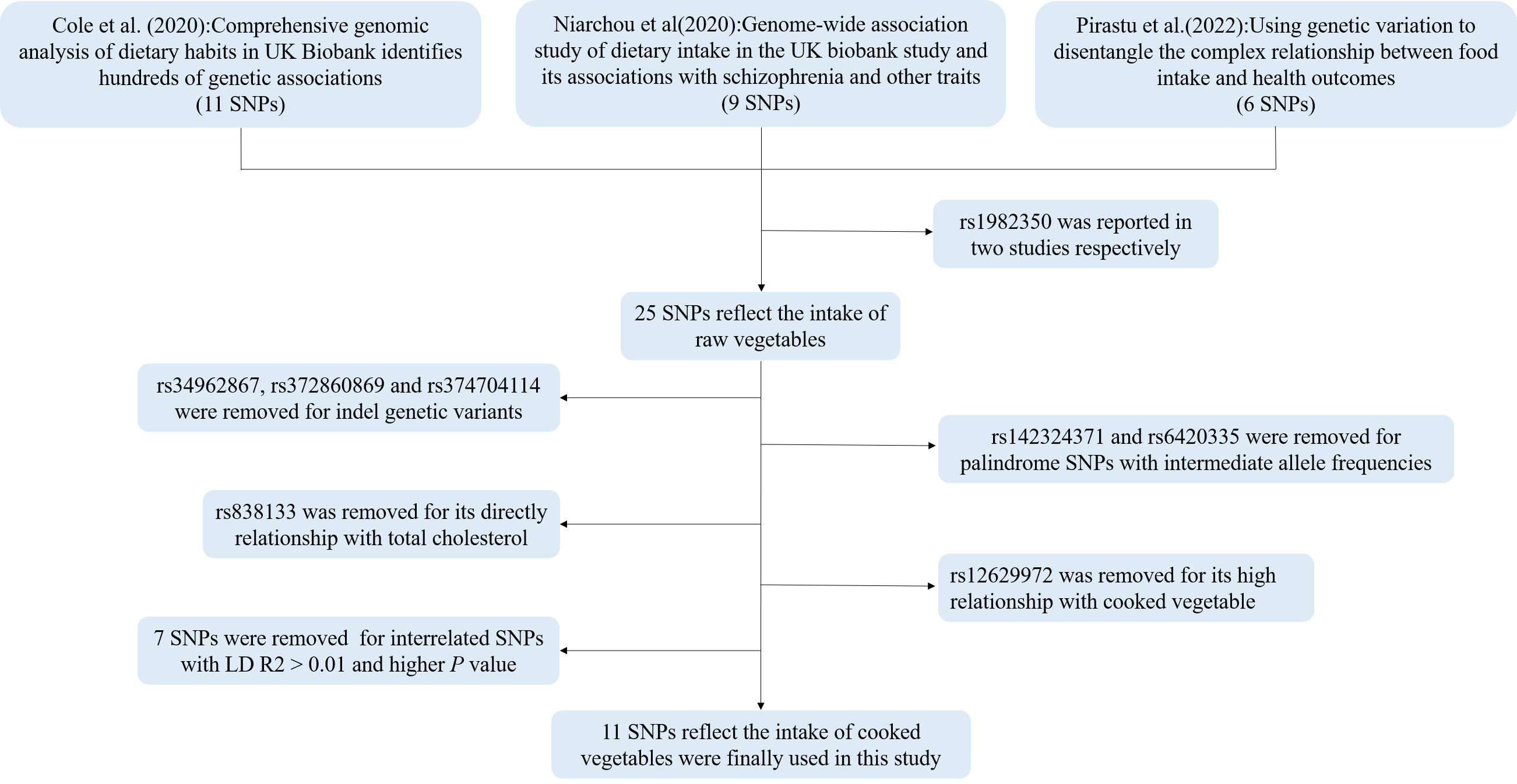
**

**Figure S2. Flowchart detailing the selection of instrument variables for cooked vegetable intake**


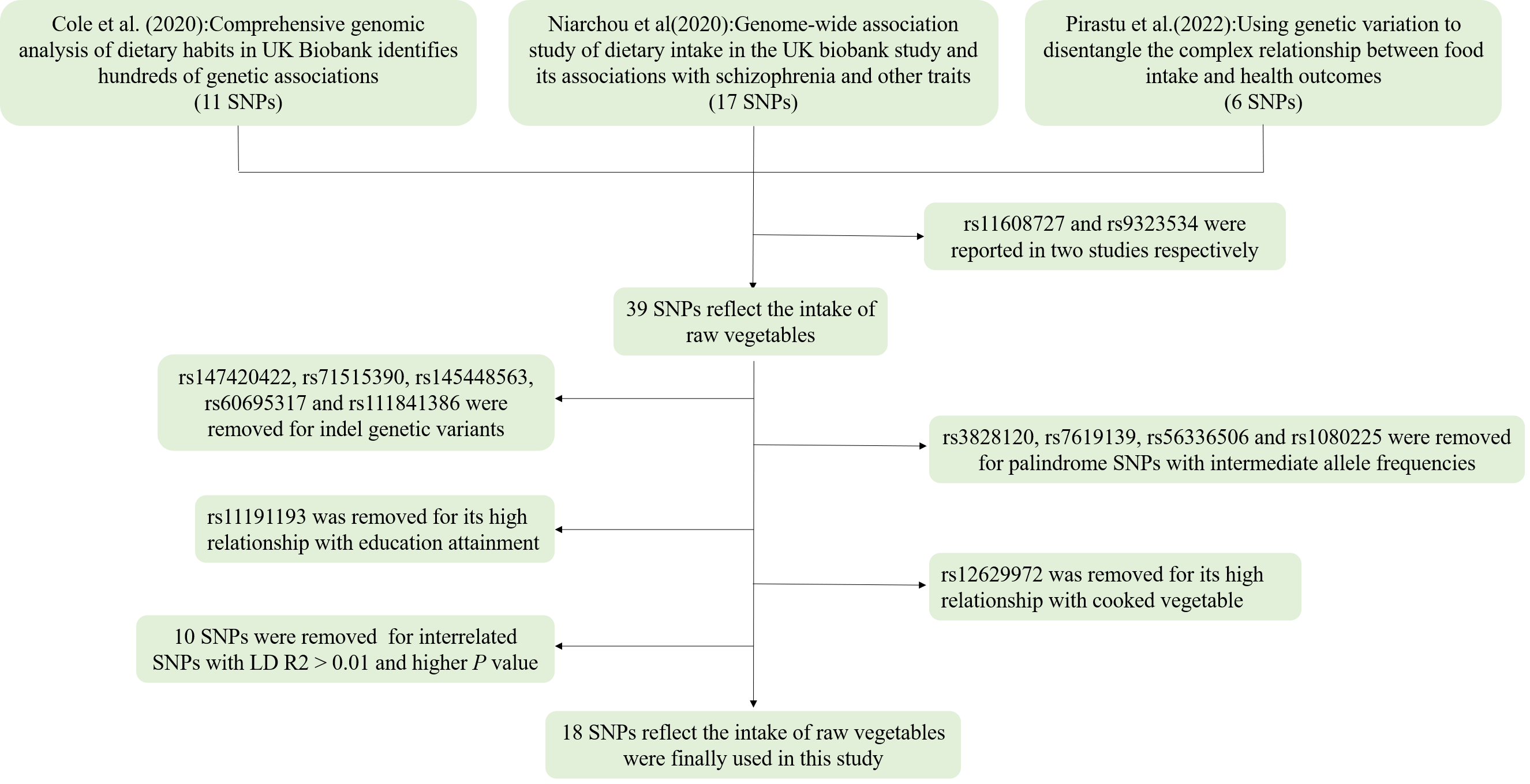


**Figure S3a. Manhattan plot of GWAS used for extracting instrument variables of raw vegetable intake in MRC-IEU(GWAS ID:ukb-b-1996)**

**
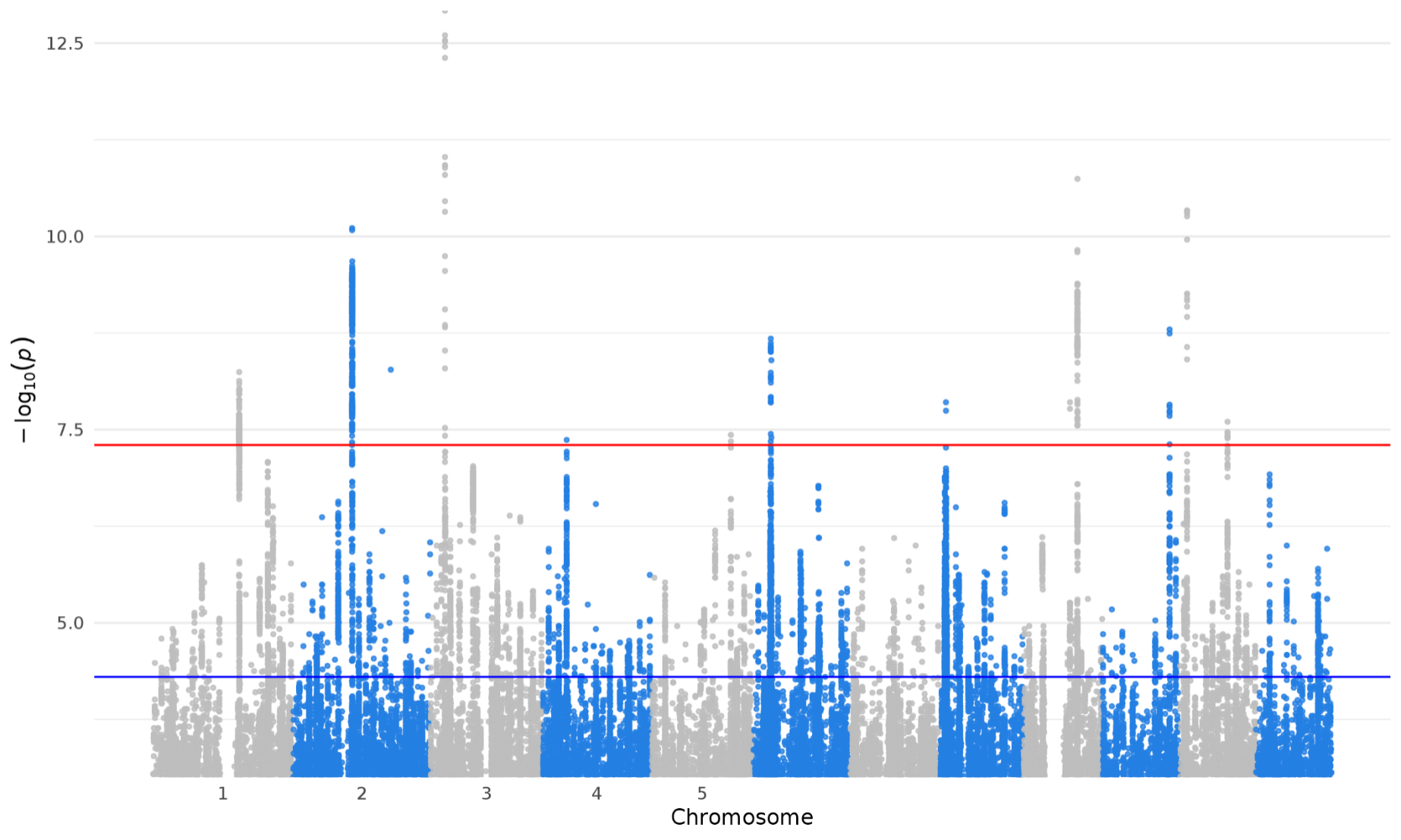
**

**Figure S3b. QQ plot of GWAS used for extracting instrument variables of raw vegetable intake in MRC-IEU(GWAS ID:ukb-b-1996)**

**
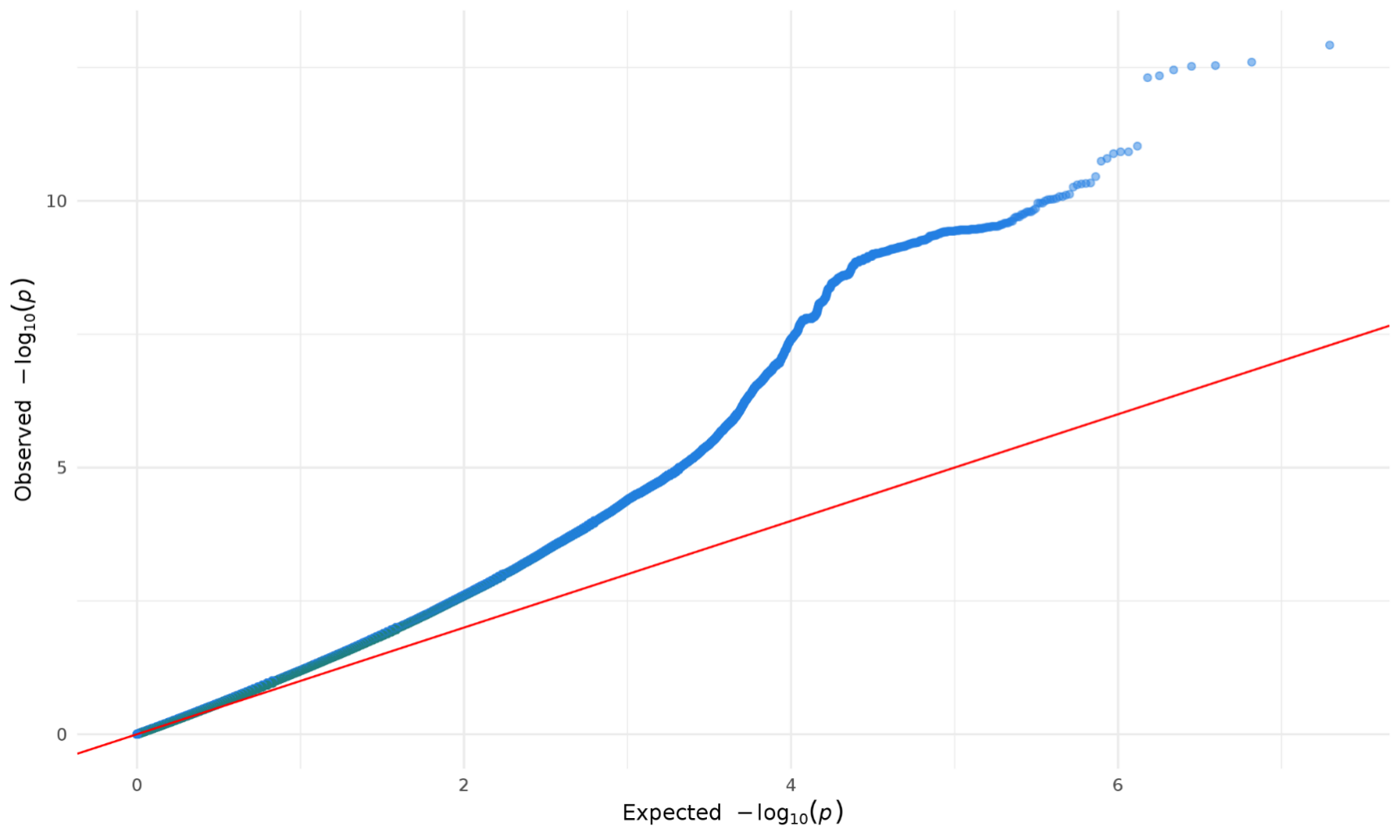
**

**Figure S4a. Manhattan plot of GWAS used for extracting instrument variables of cooked vegetable intake in MRC-IEU(GWAS ID:ukb-b-8089)**

**
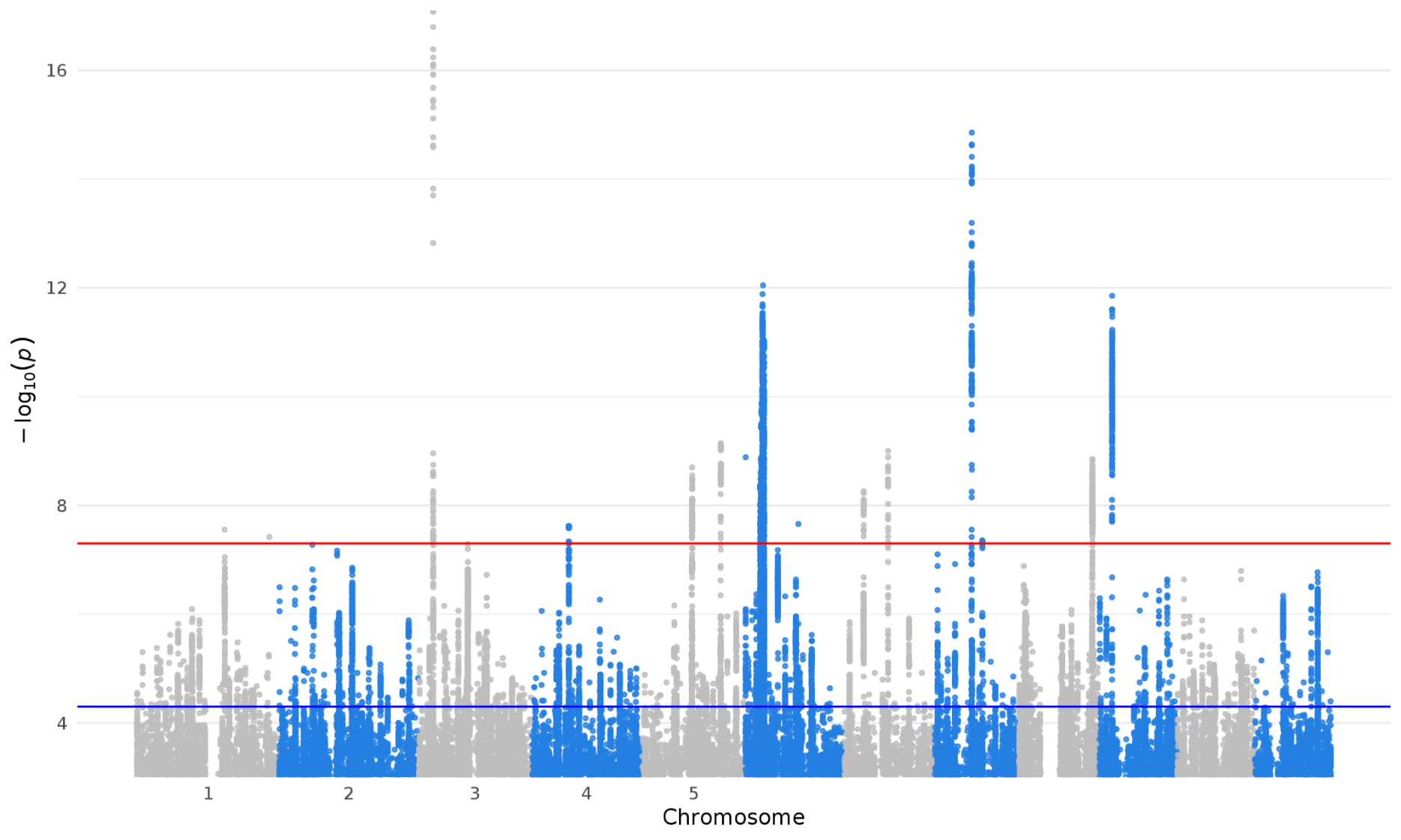
**

**Figure S4b. QQ plot of GWAS used for extracting instrument variables of cooked vegetable intake in MRC-IEU(GWAS ID:ukb-b-8089)**

**
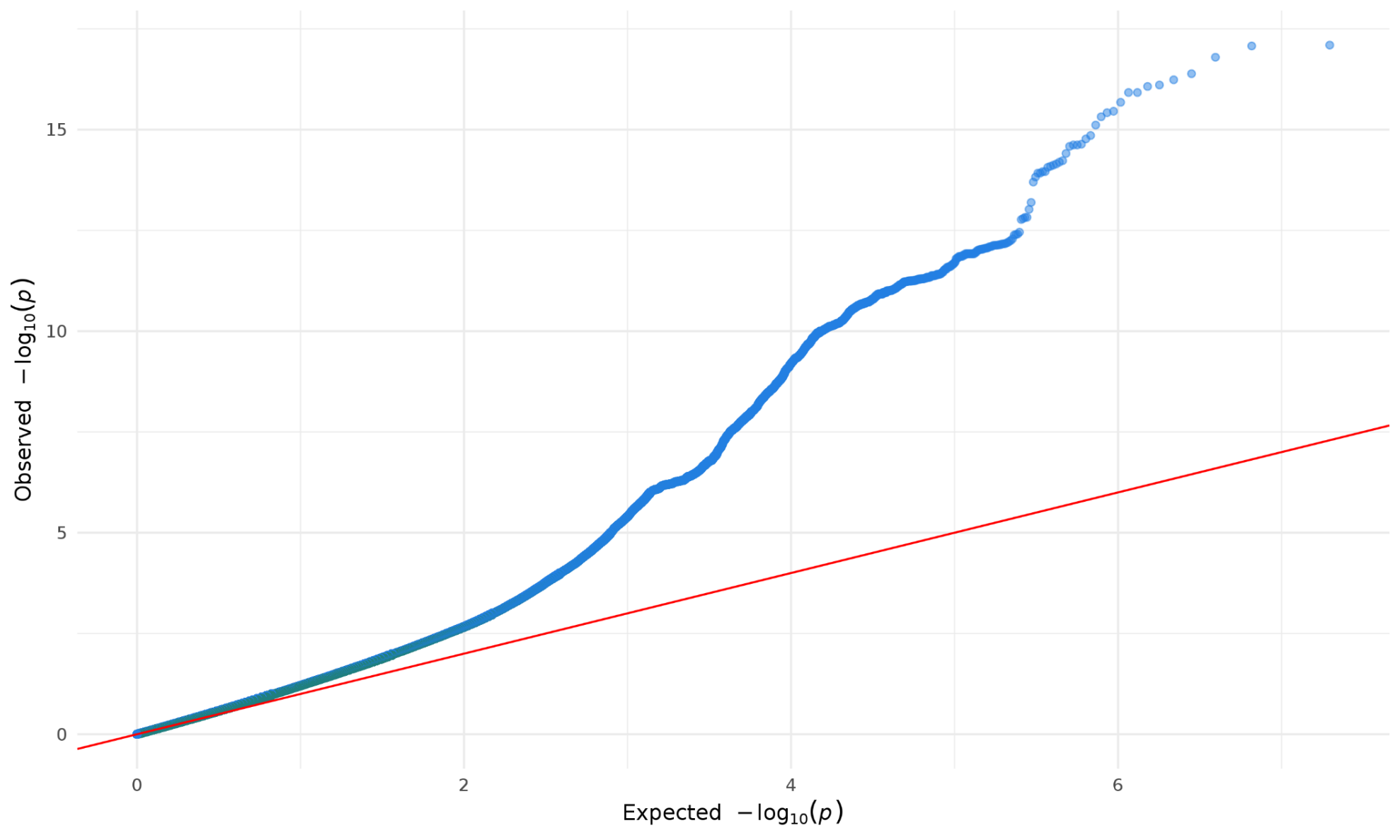
**

**Figure S5. Leave-one-out sensitivity analyses for raw vegetable intake (IV from UK biobank) on ischemic cardio-cerebral vascular diseases**

**(A)Angina pectoris**

**
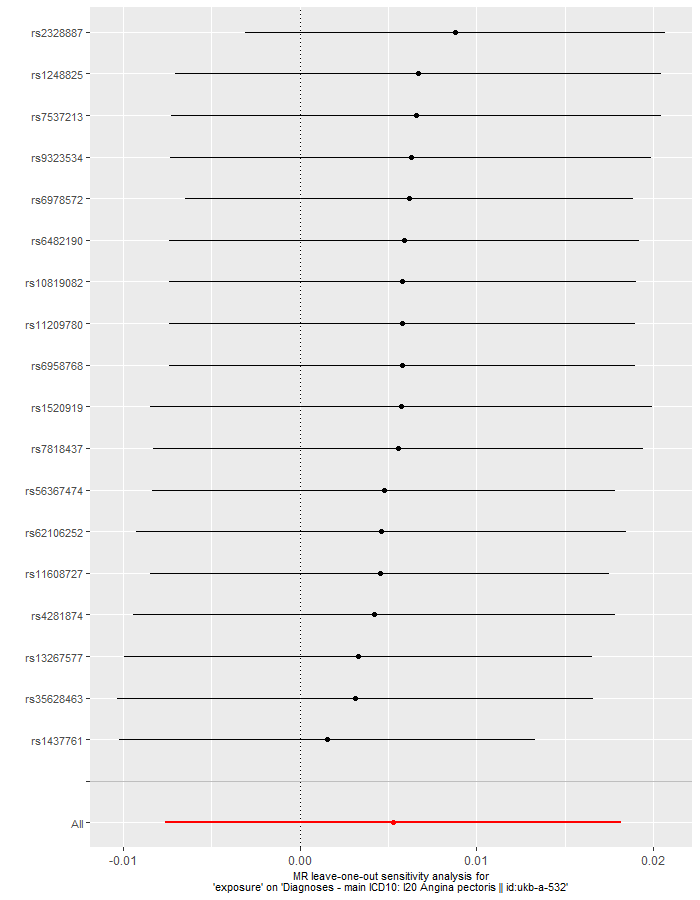
**

**(B)Acute myocardial infarction**

**
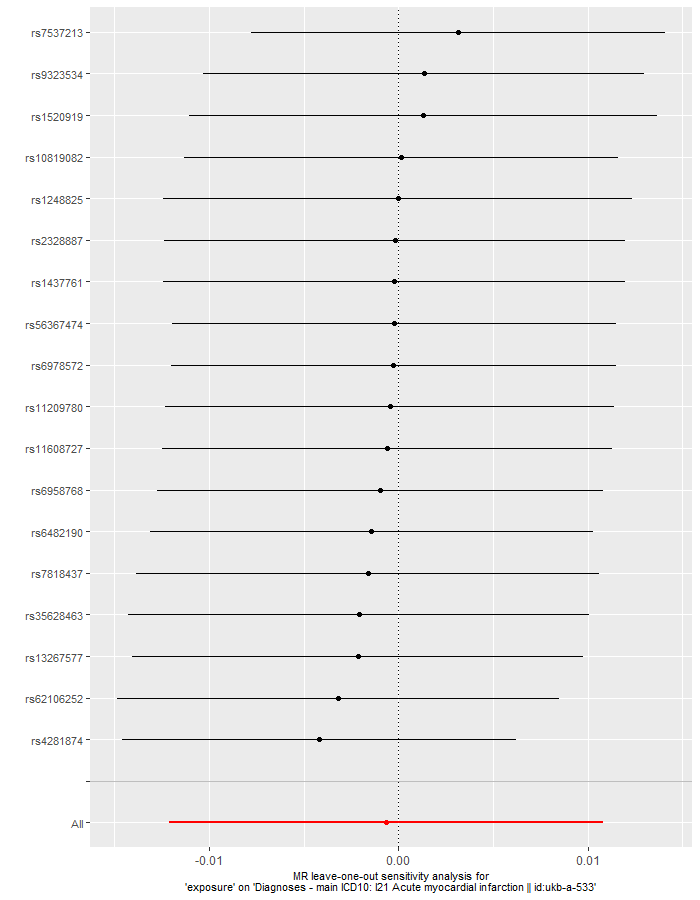
**

**(C)Chronic ischemic heart disease**


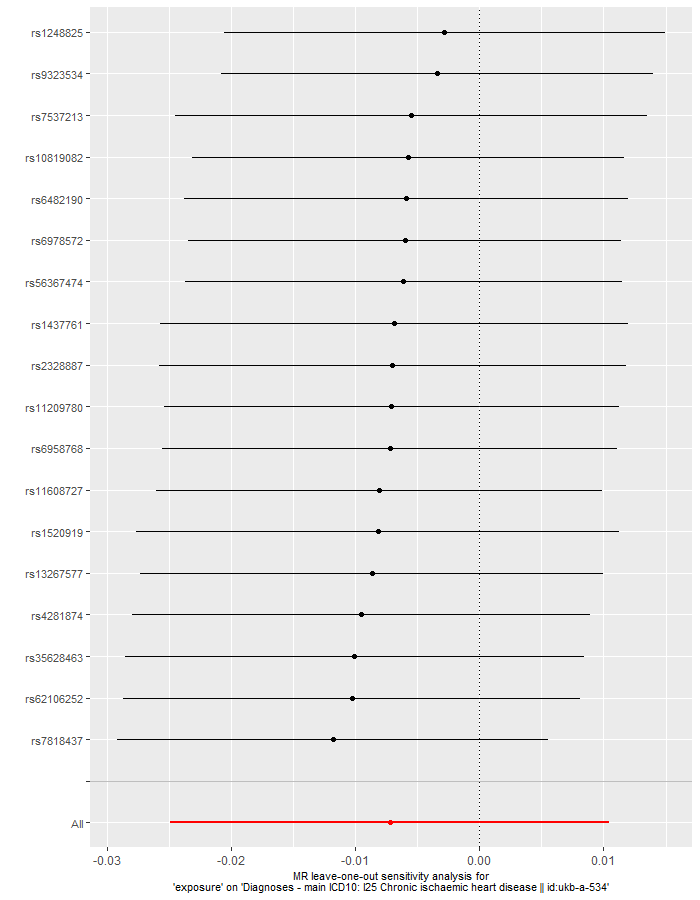


**(D)Cerebral infarction**


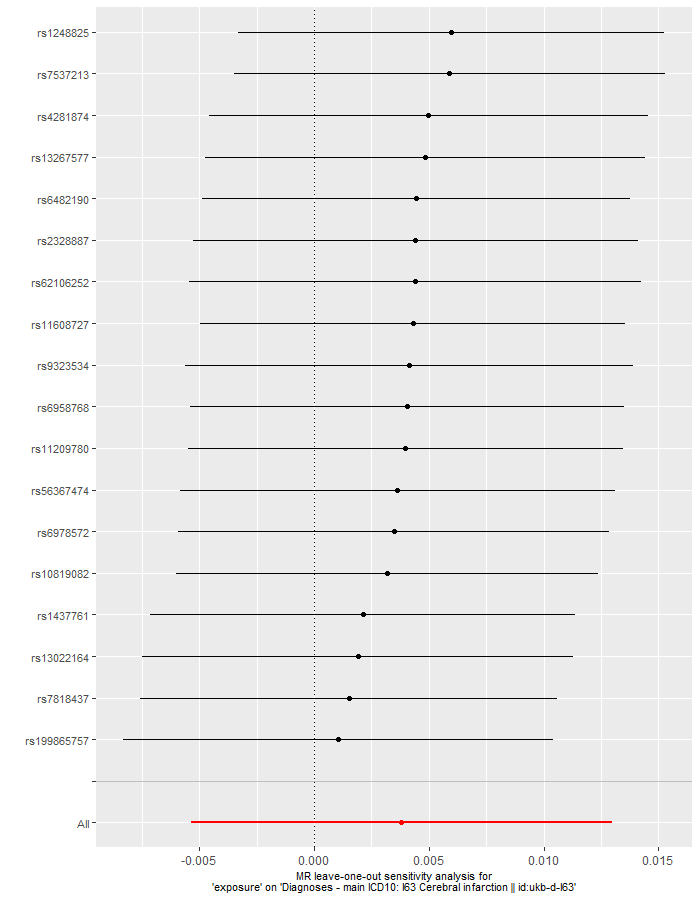


**Figure S6. Leave-one-out sensitivity analyses for cooked vegetable intake (IV from UK biobank) on ischemic cardio-cerebral vascular diseases**

**(A)Angina pectoris**


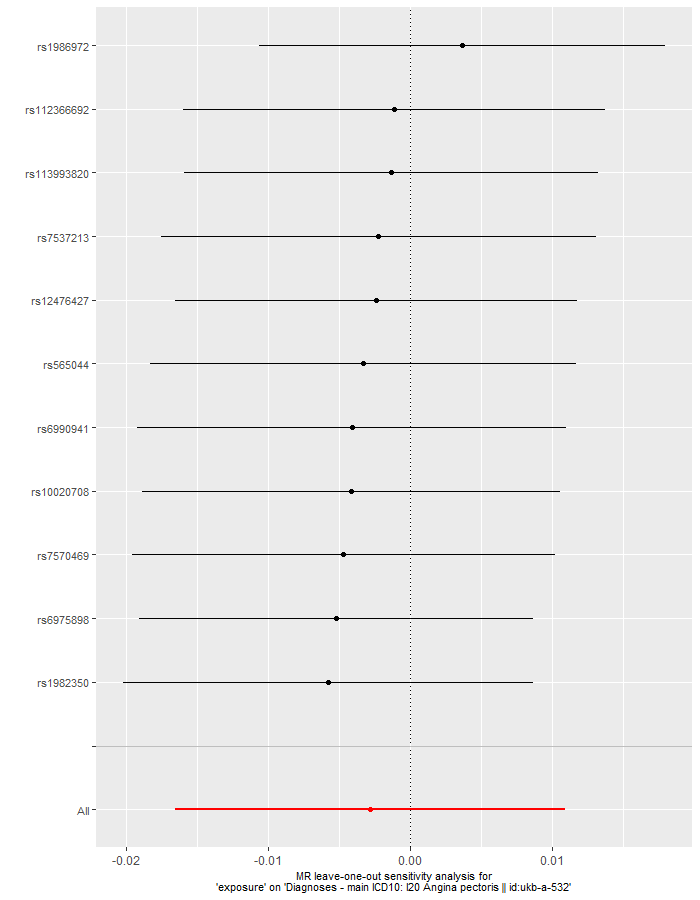


**(B)Acute myocardial infarction**


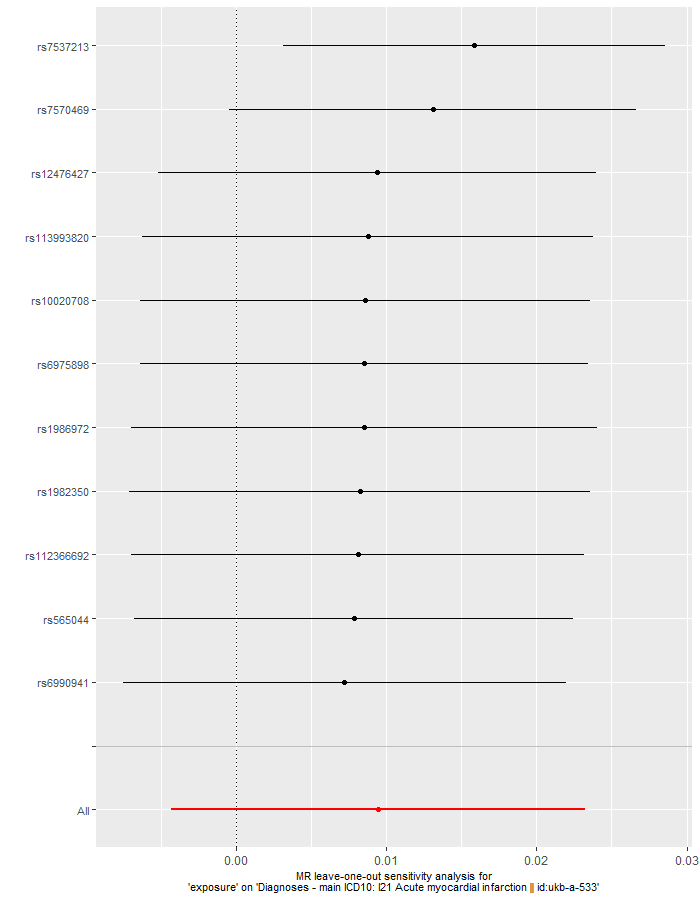


**(C)Chronic ischemic heart disease**


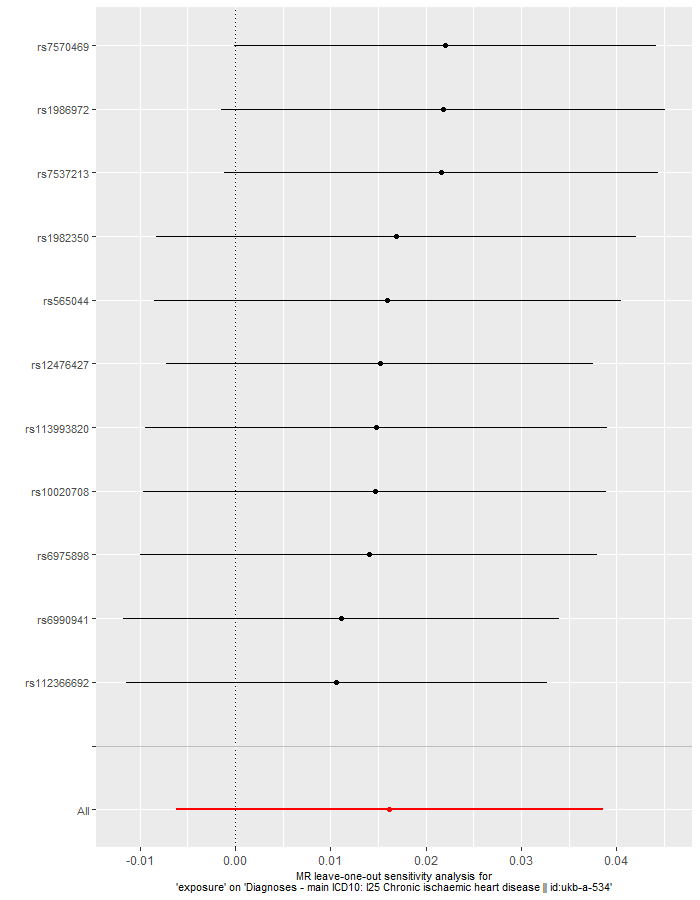


**(D)Cerebral infarction**


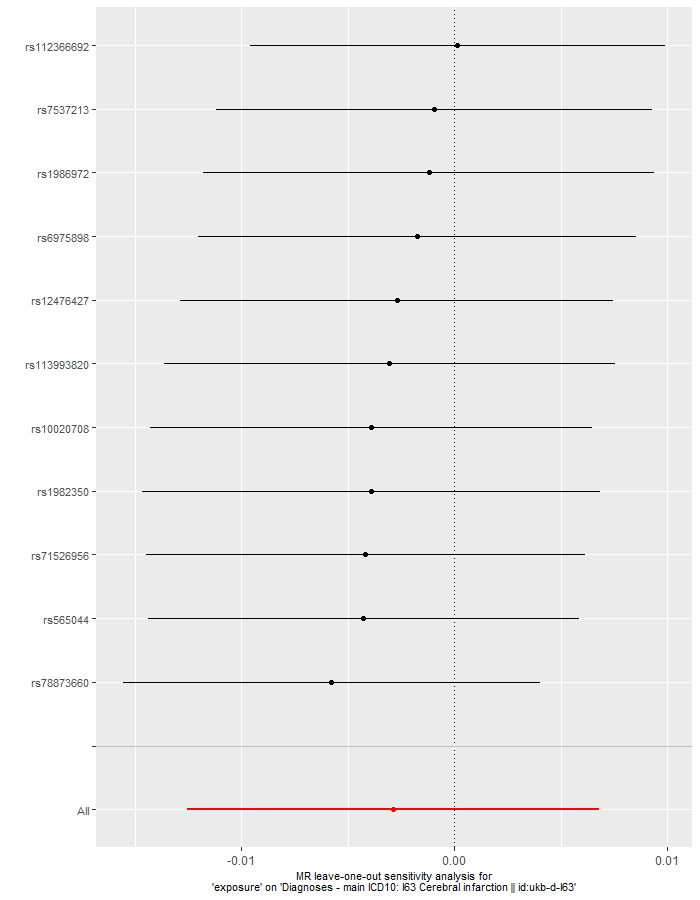


**Figure S7. Leave-one-out sensitivity analyses for raw vegetable intake (IV from UK biobank) on lipid profile**

**(A)Apolipoprotein A1**


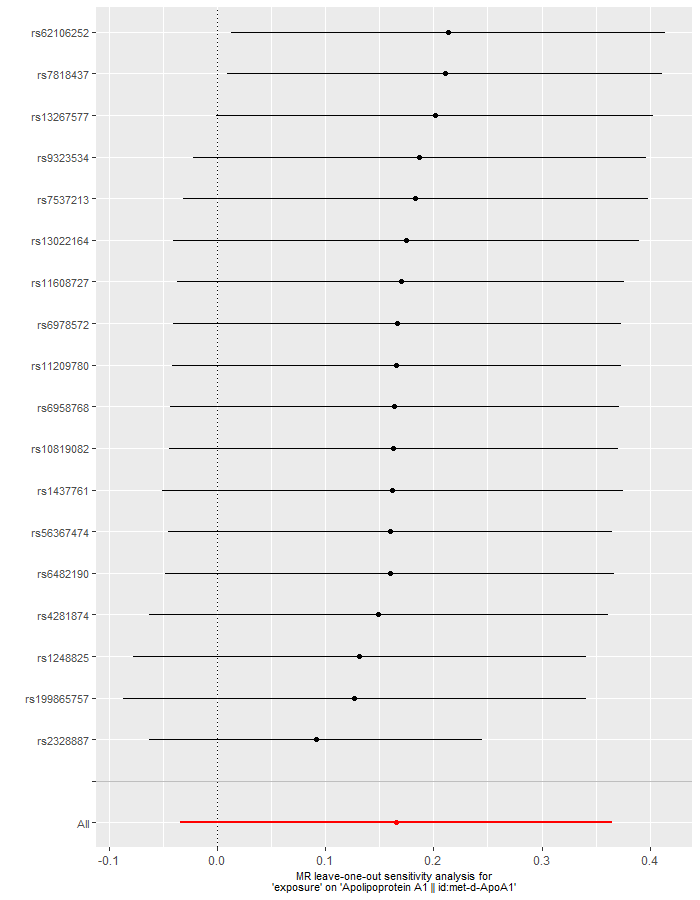


**(B)Apolipoprotein B**


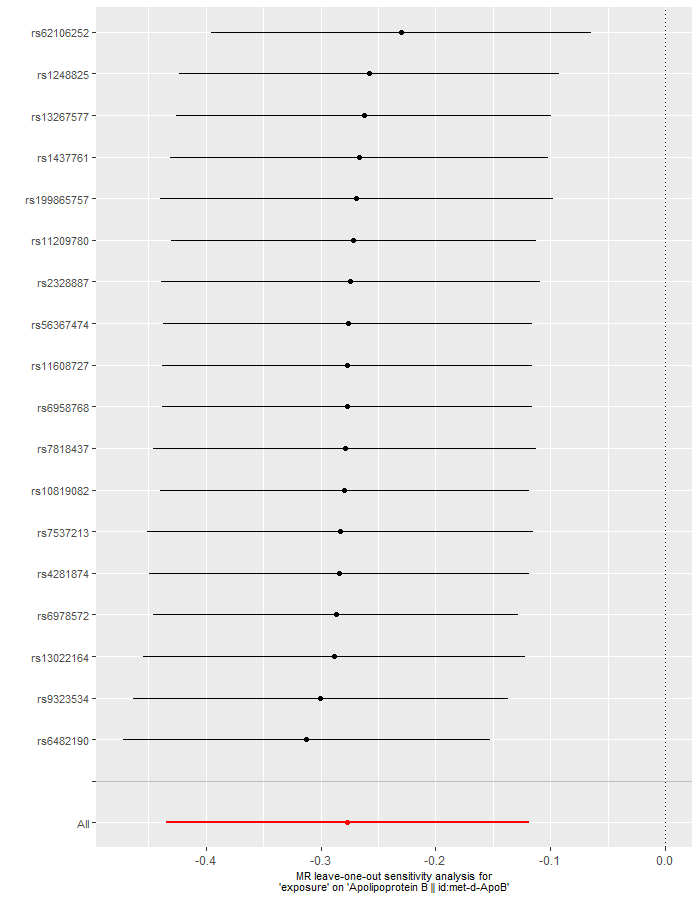


**(C)HDL cholesterol**


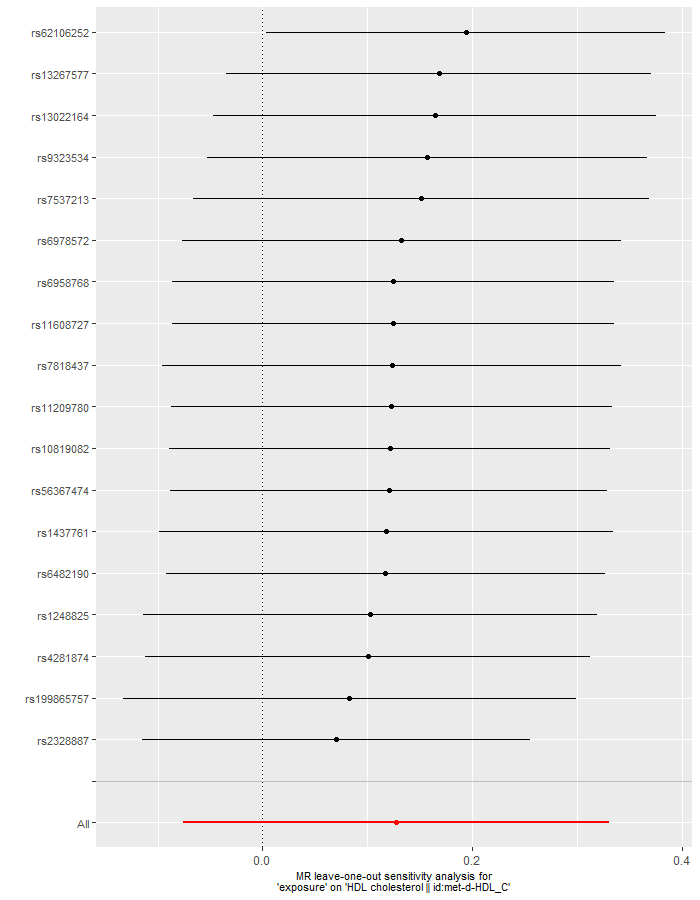


**(D)LDL cholesterol**


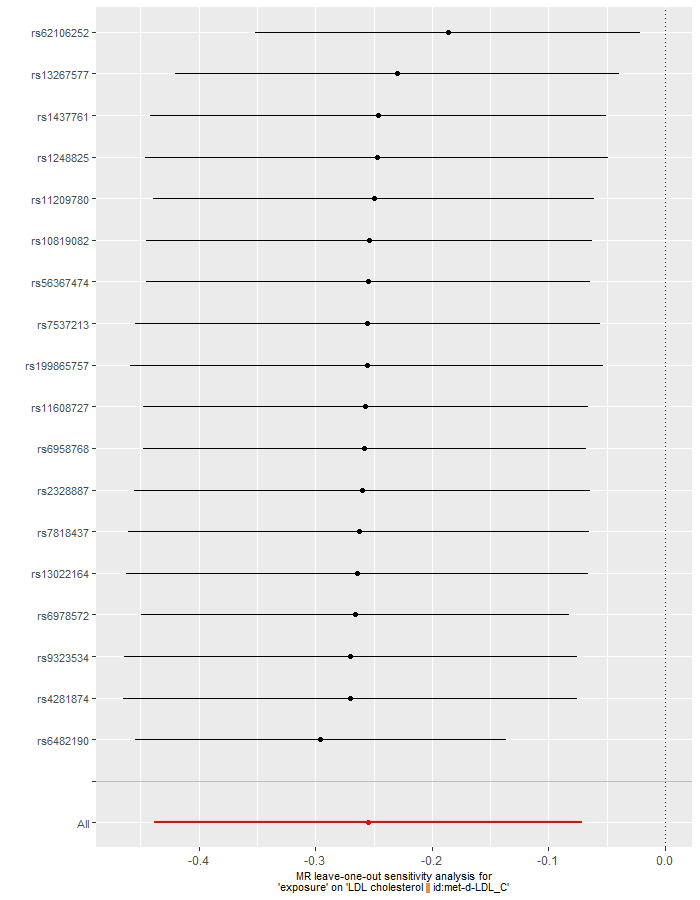


**(E)Total triglycerides**


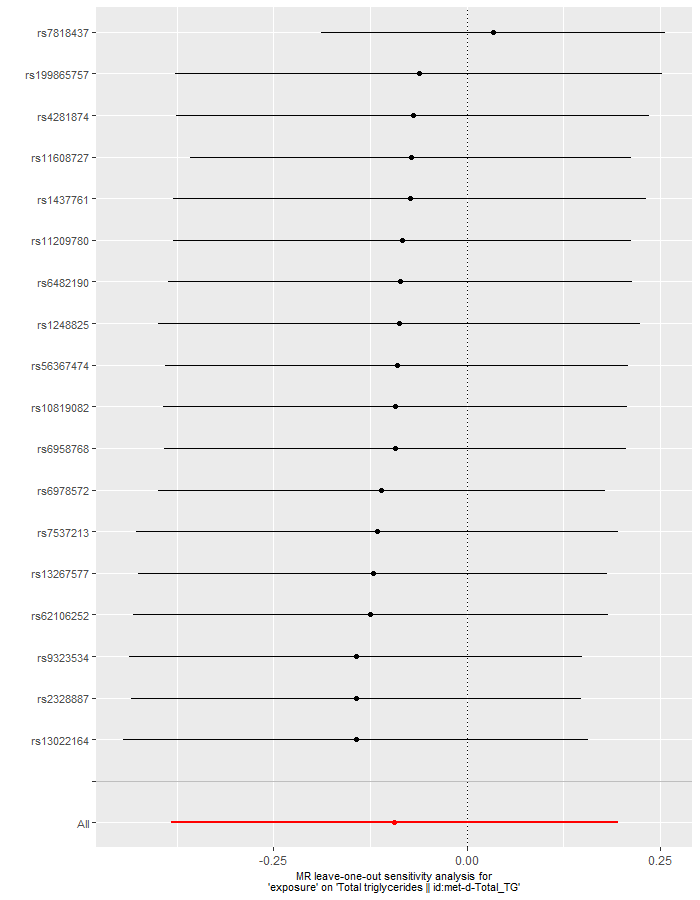


**(F)Total cholesterol**


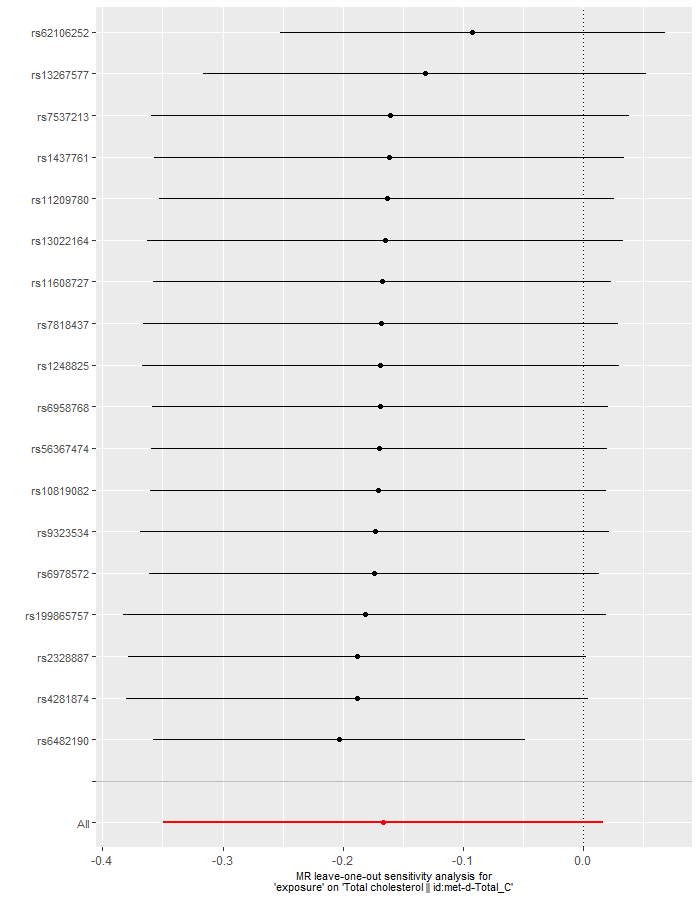


**Figure S8. Leave-one-out sensitivity analyses for cooked vegetable intake (IV from UK biobank) on lipid profile**

**(A)Apolipoprotein A1**


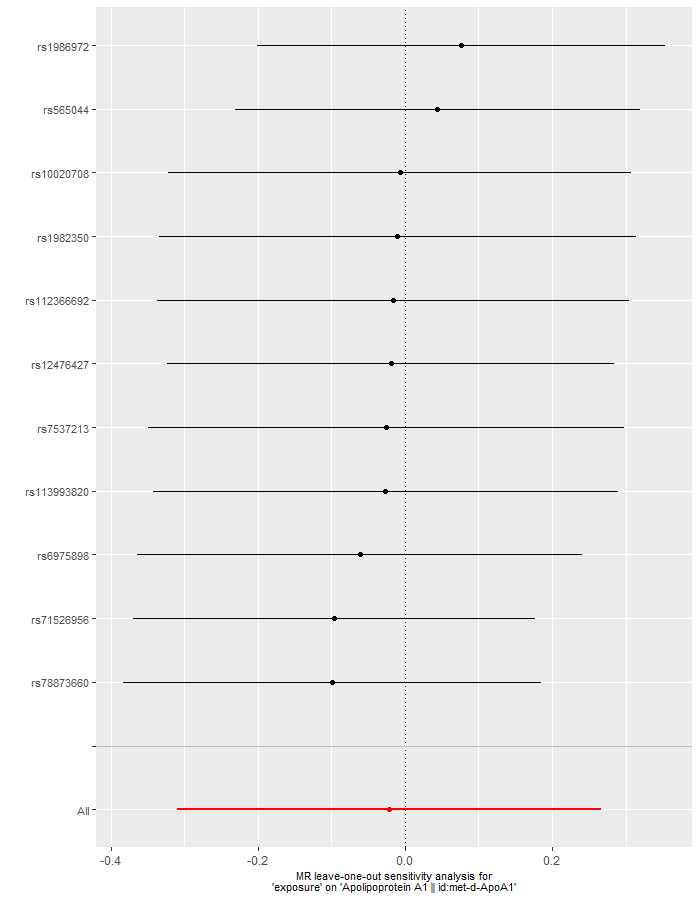


**(B)Apolipoprotein B**


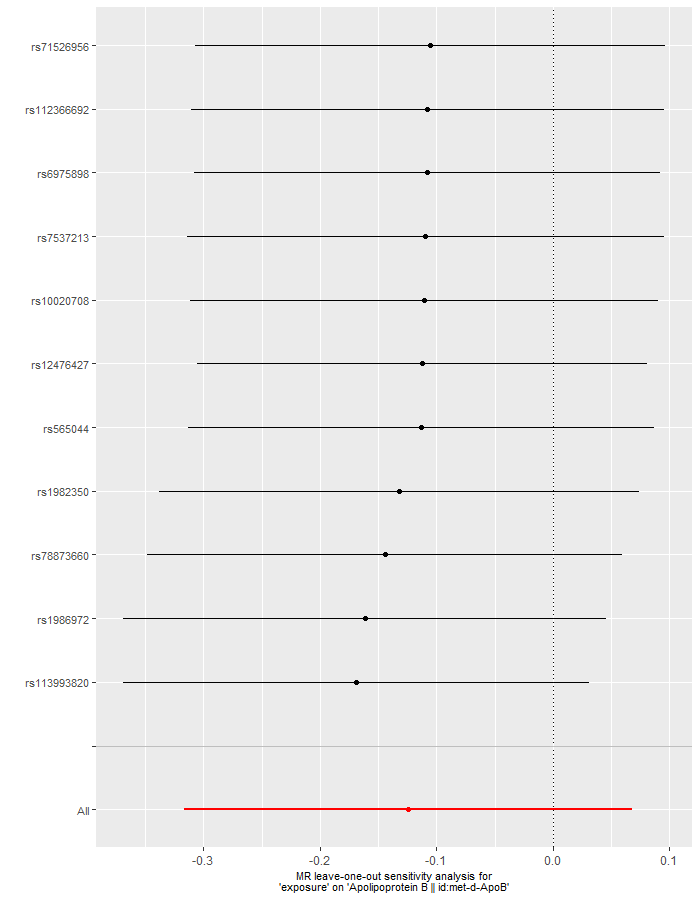


**(C)HDL cholesterol**


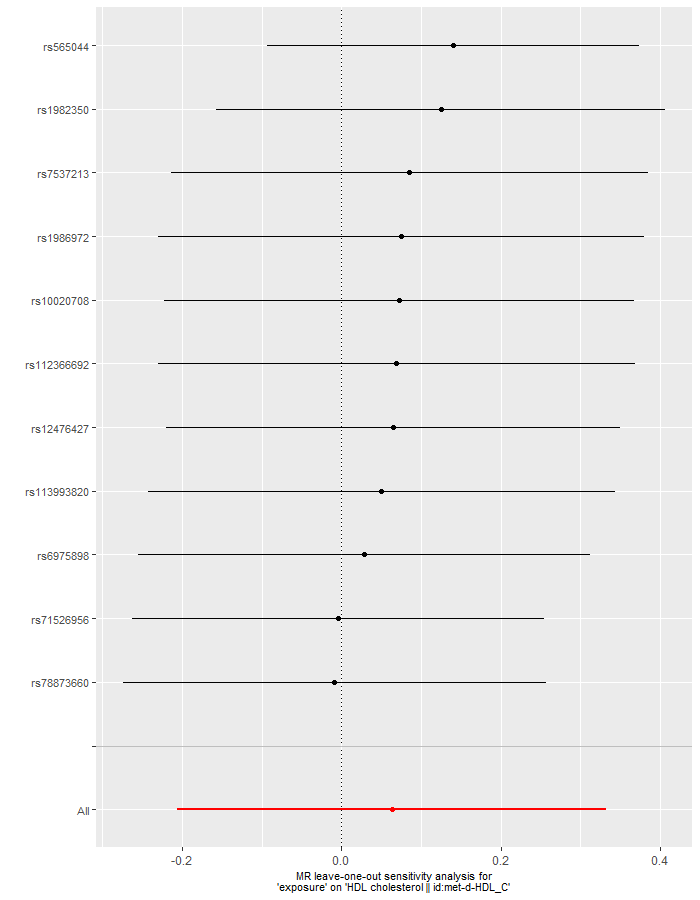


**(D)LDL cholesterol**


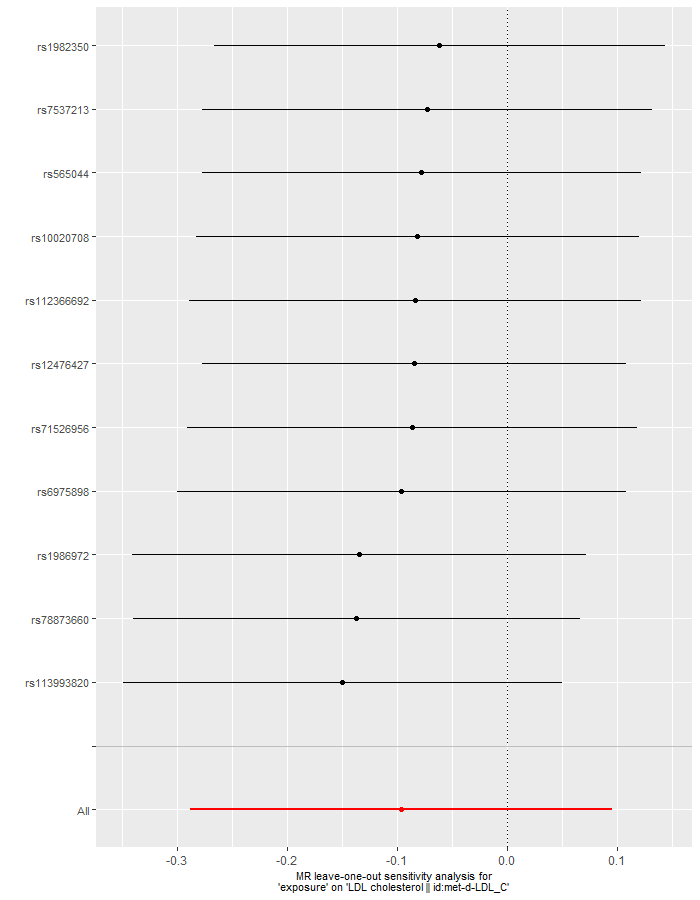


**(E)Total triglycerides**


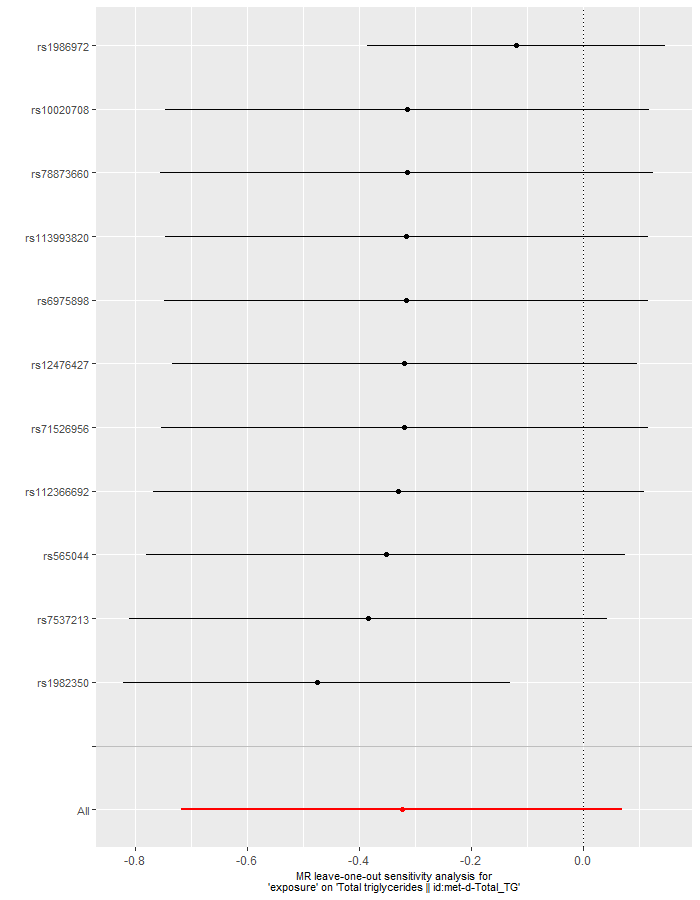


**(F)Total cholesterol**


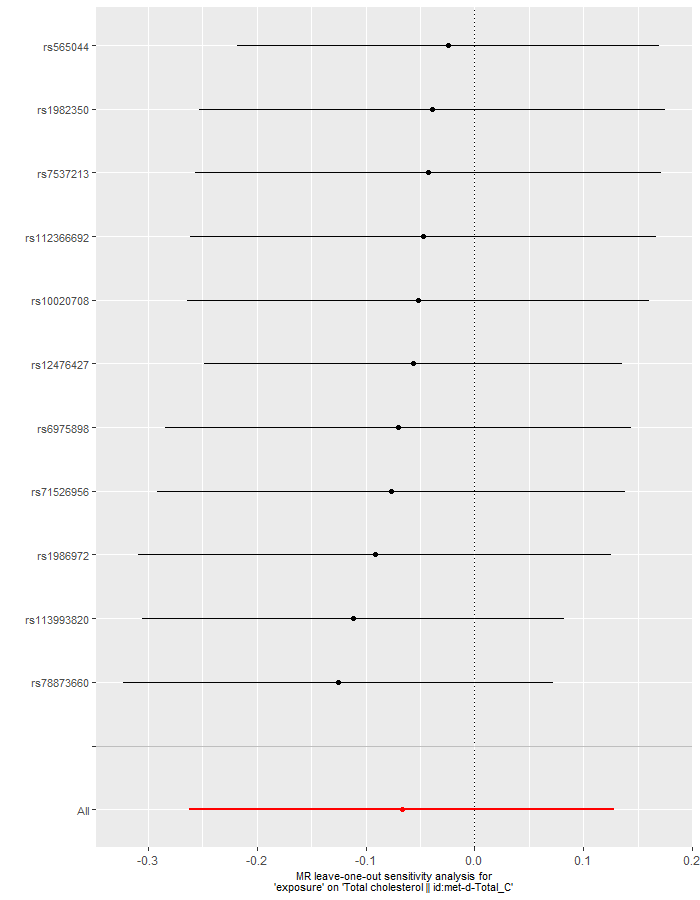


**Figure S9. Leave-one-out sensitivity analyses for raw vegetable intake (IV from MRC-IEU) on ischemic cardio-cerebral vascular diseases**

**(A)Angina pectoris**

**
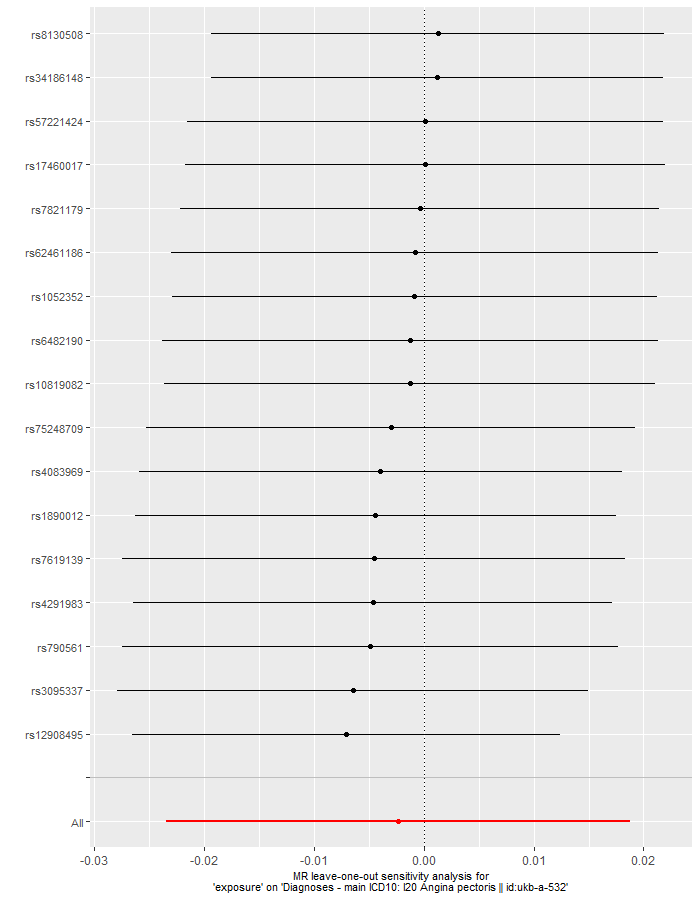
**

**(B)Acute myocardial infarction**

**
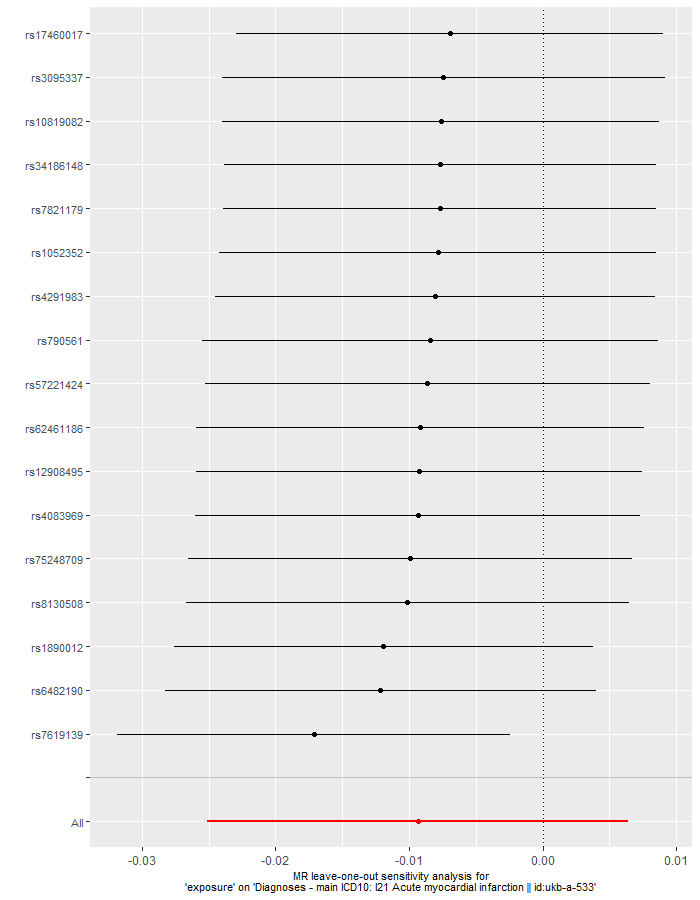
**

**(C)Chronic ischemic heart disease**


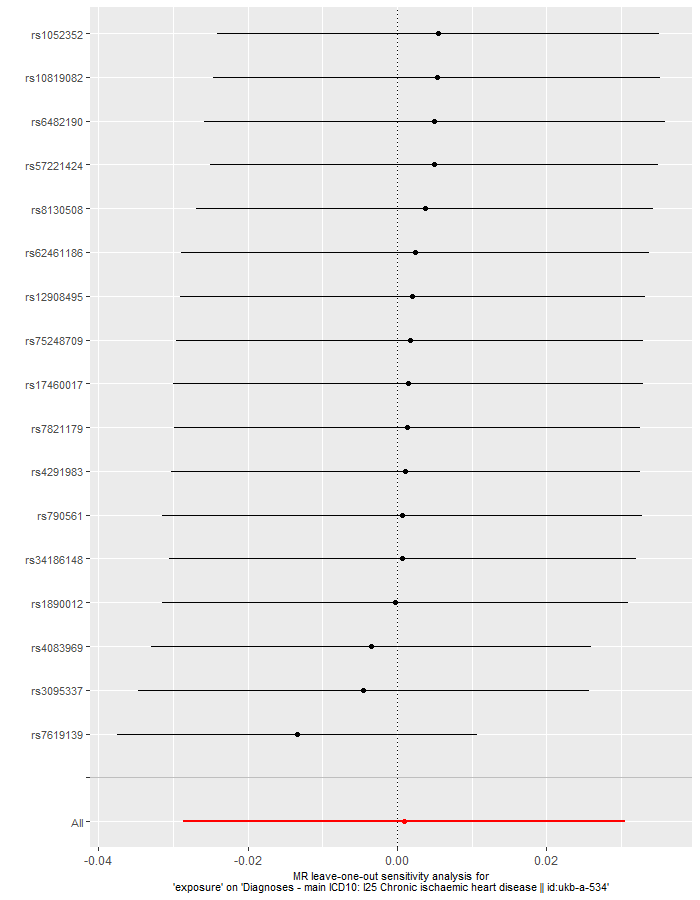


**(D)Cerebral infarction**


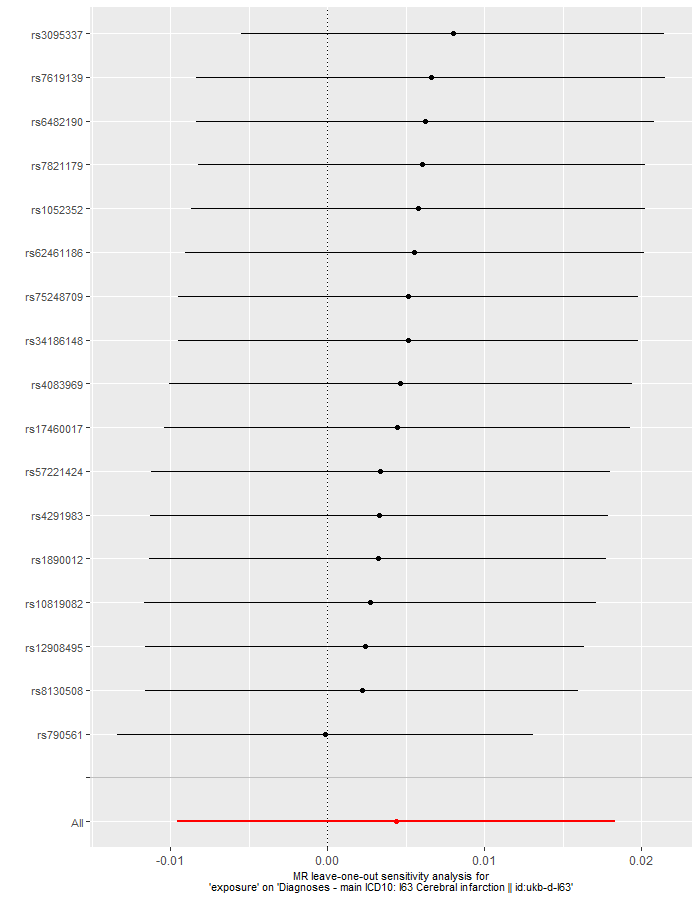


**Figure S10. Leave-one-out sensitivity analyses for cooked vegetable intake (IV from MRC-IEU) on ischemic cardio-cerebral vascular diseases**

**(A)Angina pectoris**


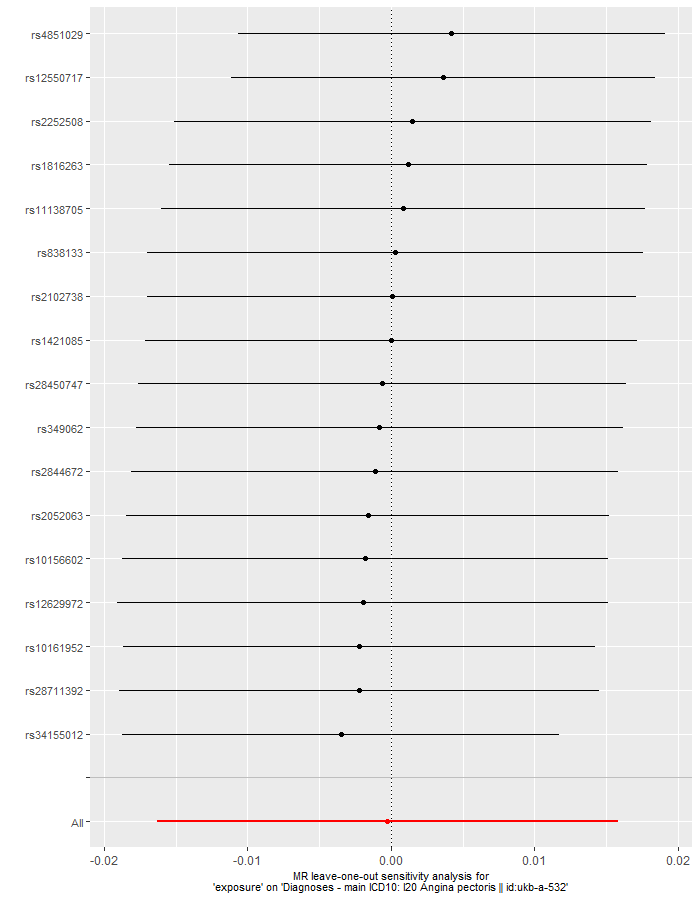


**(B)Acute myocardial infarction**


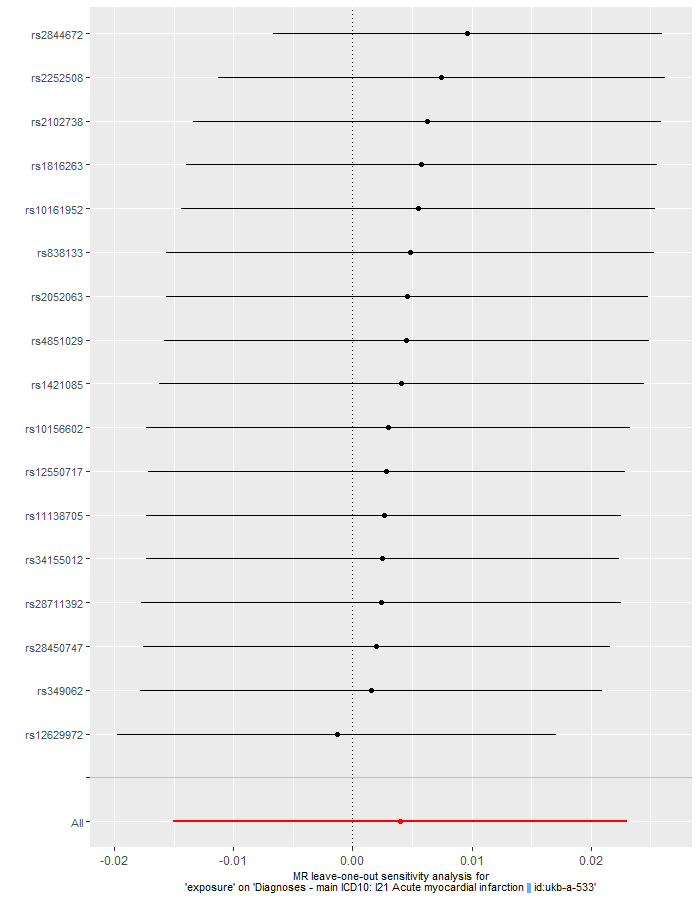


**(C)Chronic ischemic heart disease**


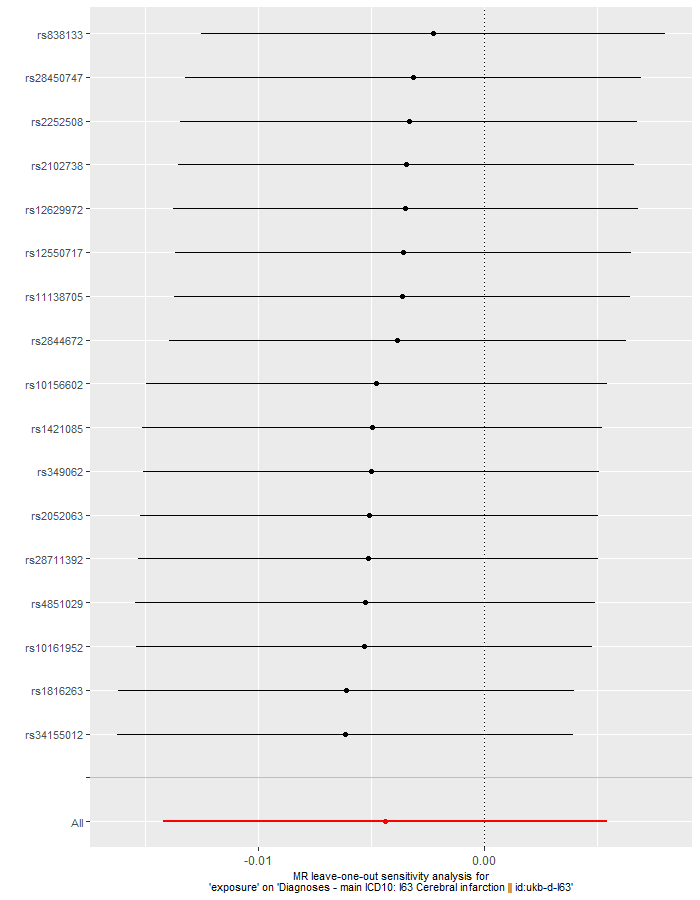


**(D)Cerebral infarction**

**
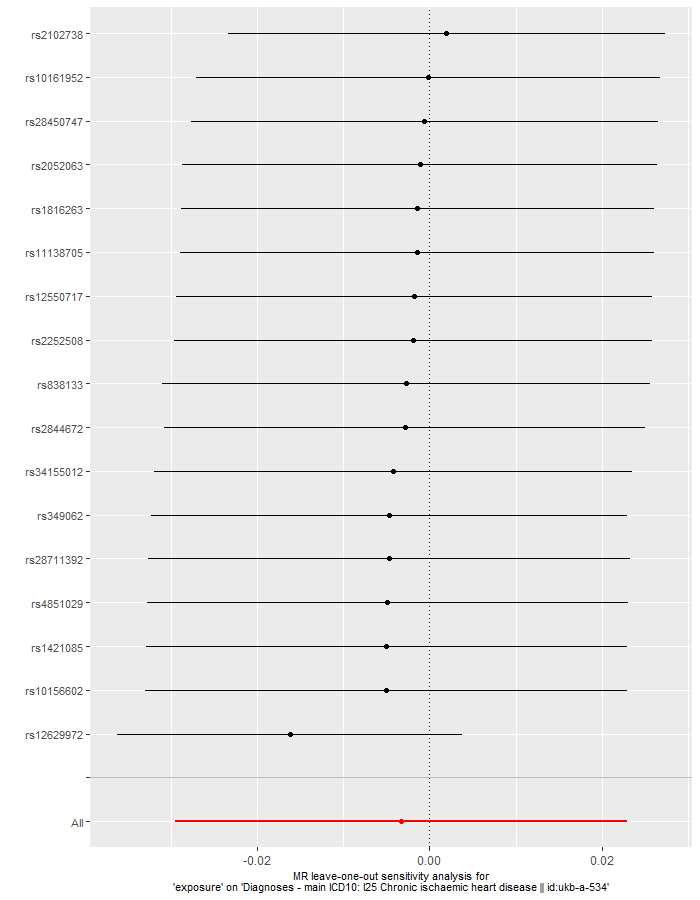
**

**Figure S11. Leave-one-out sensitivity analyses for raw vegetable intake (IV from MRC-IEU) on lipid profile**

**(A)Apolipoprotein A1**


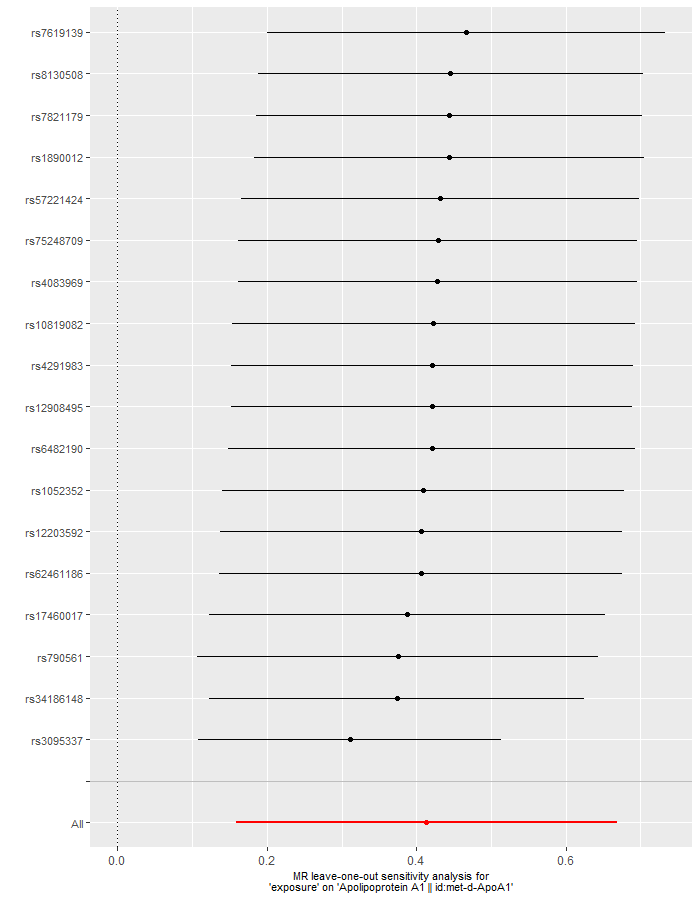


**(B)Apolipoprotein B**


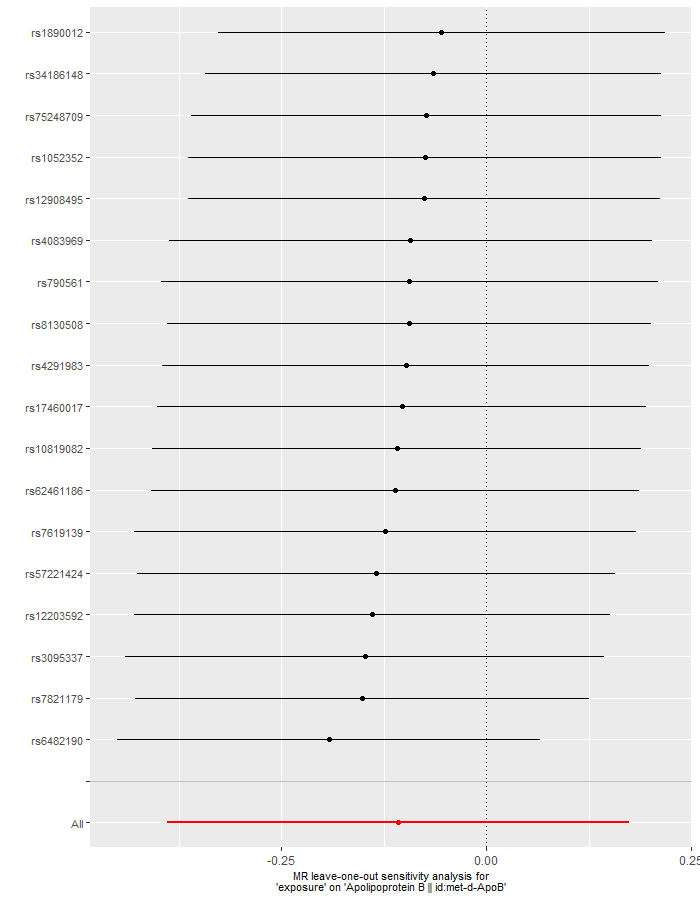


**(C)HDL cholesterol**


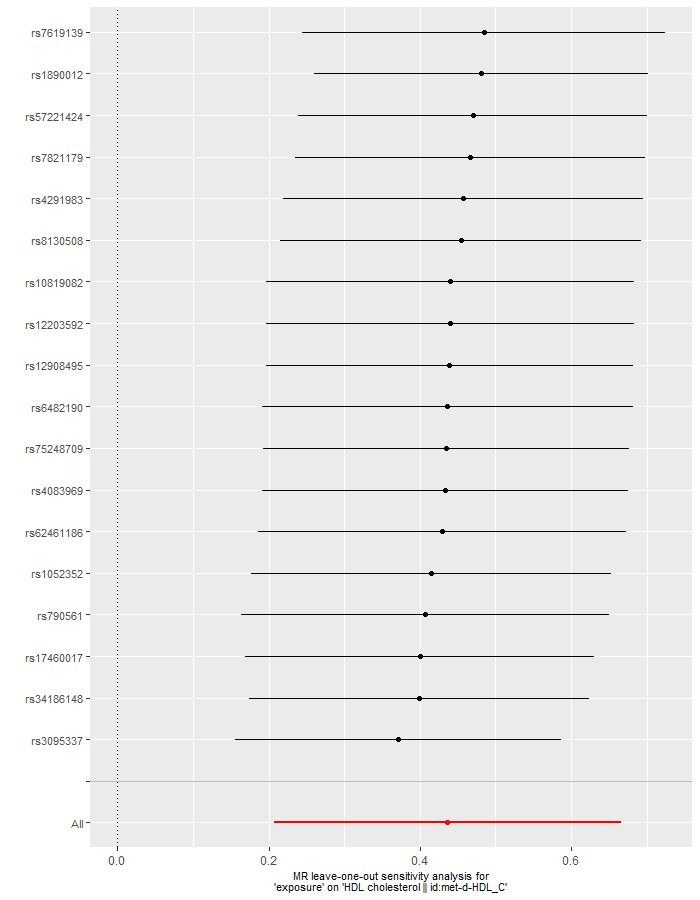


**(D)LDL cholesterol**


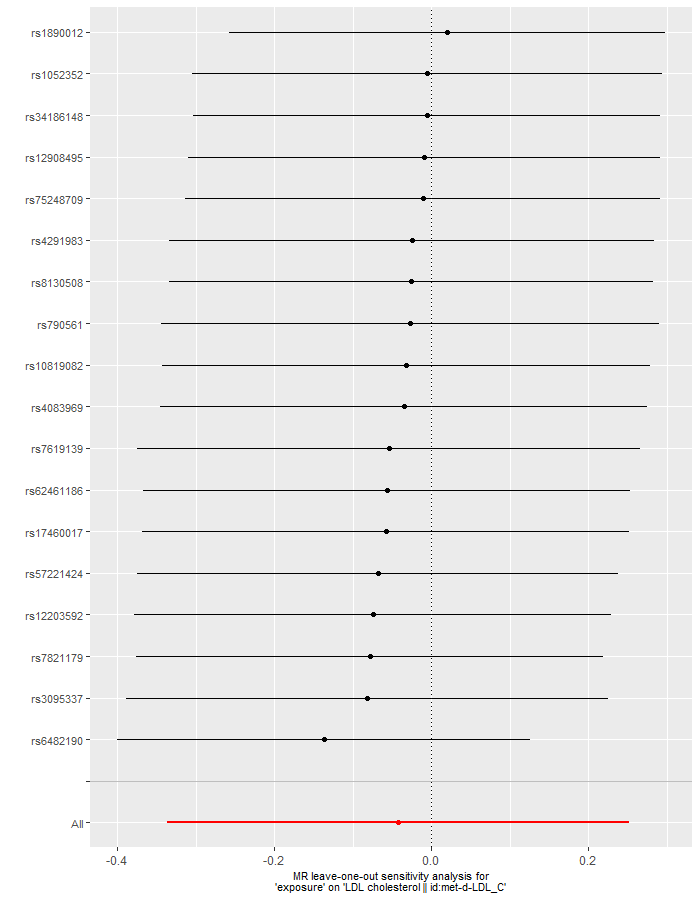


**(E)Total triglycerides**


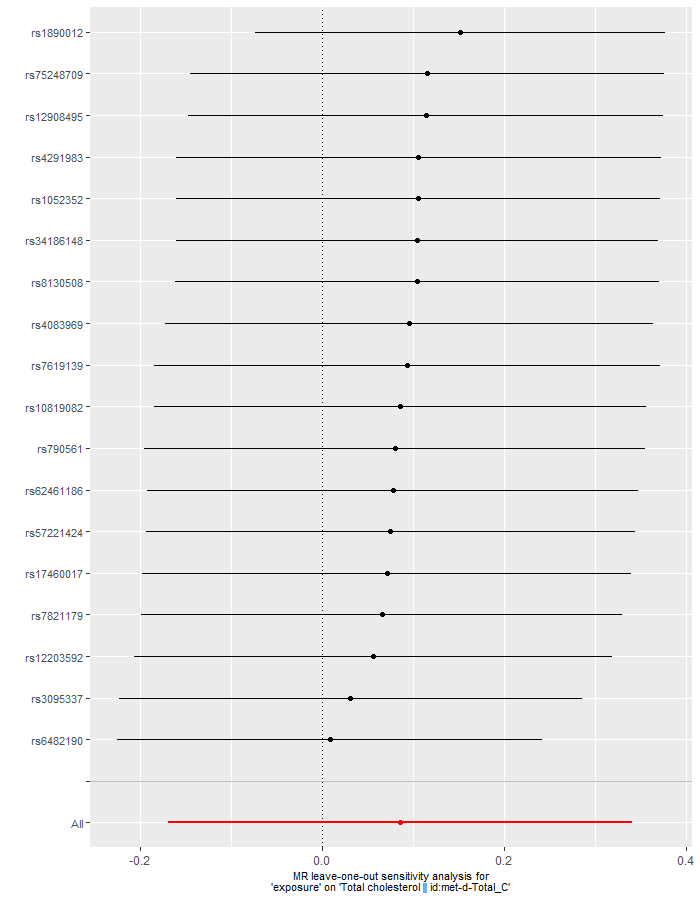


**(F)Total cholesterol**


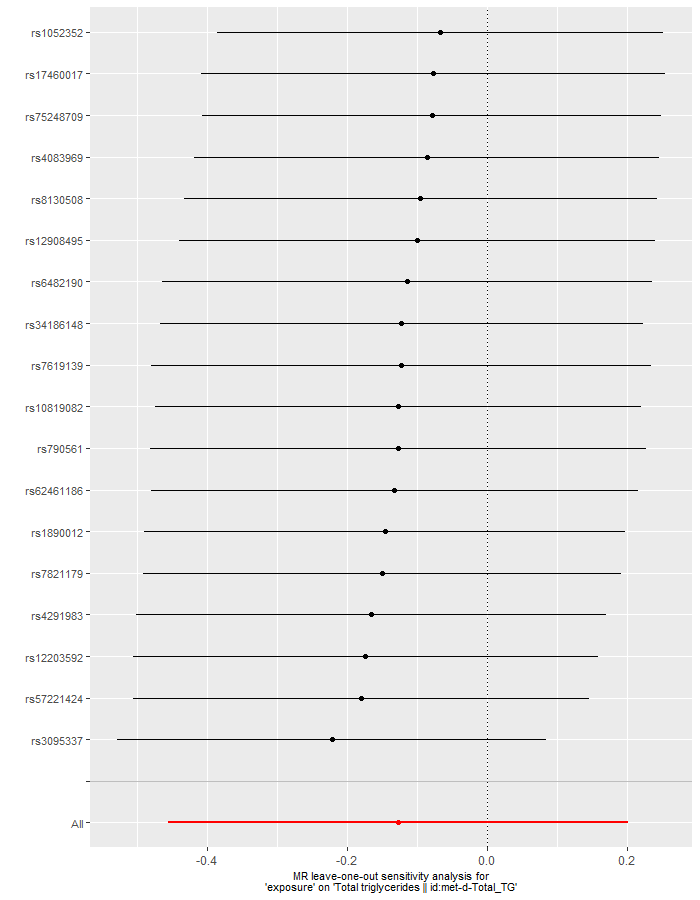


**Figure S12. Leave-one-out sensitivity analyses for cooked vegetable intake (IV from MRC-IEU) on lipid profile**

**(A)Apolipoprotein A1**


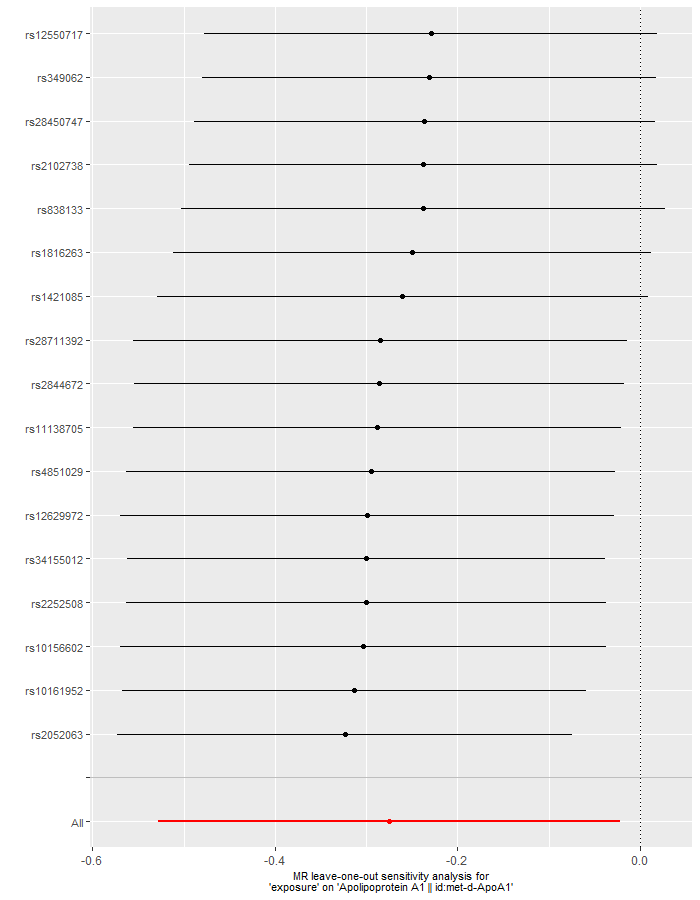


**(B)Apolipoprotein B**


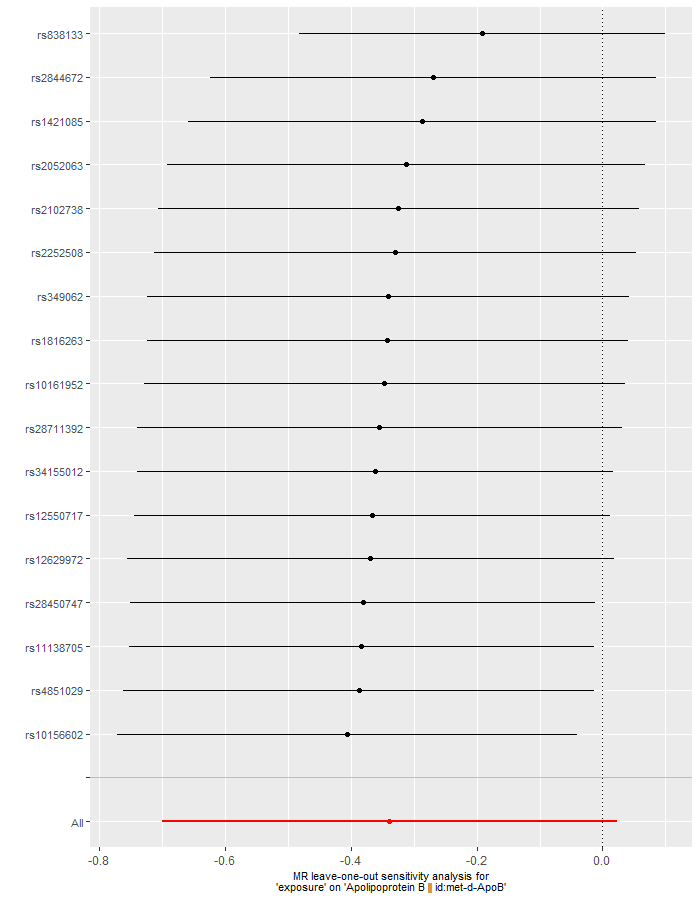


**(C)HDL cholesterol**

**
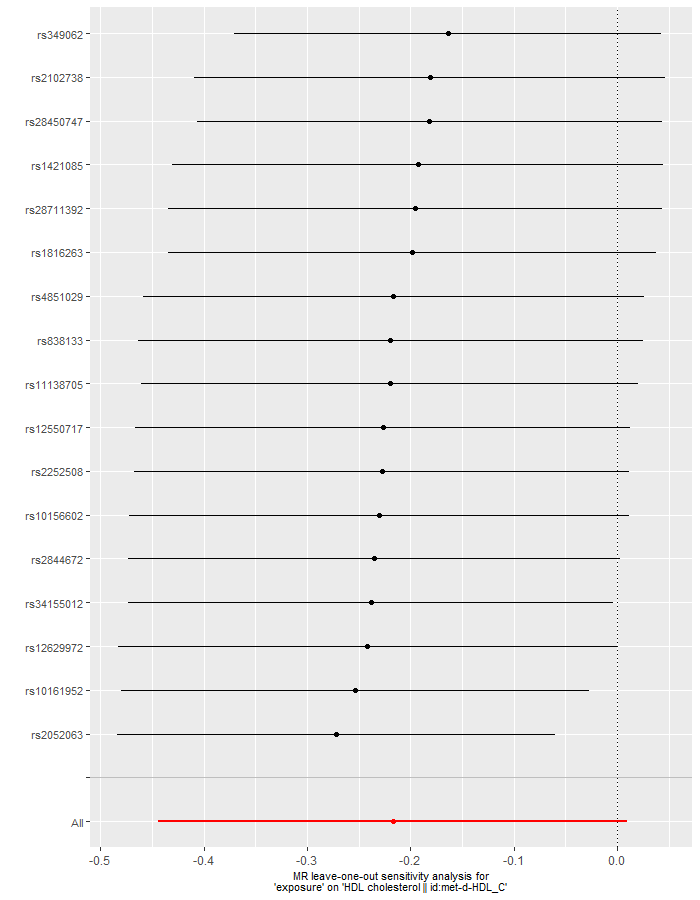
**

**(D)LDL cholesterol**


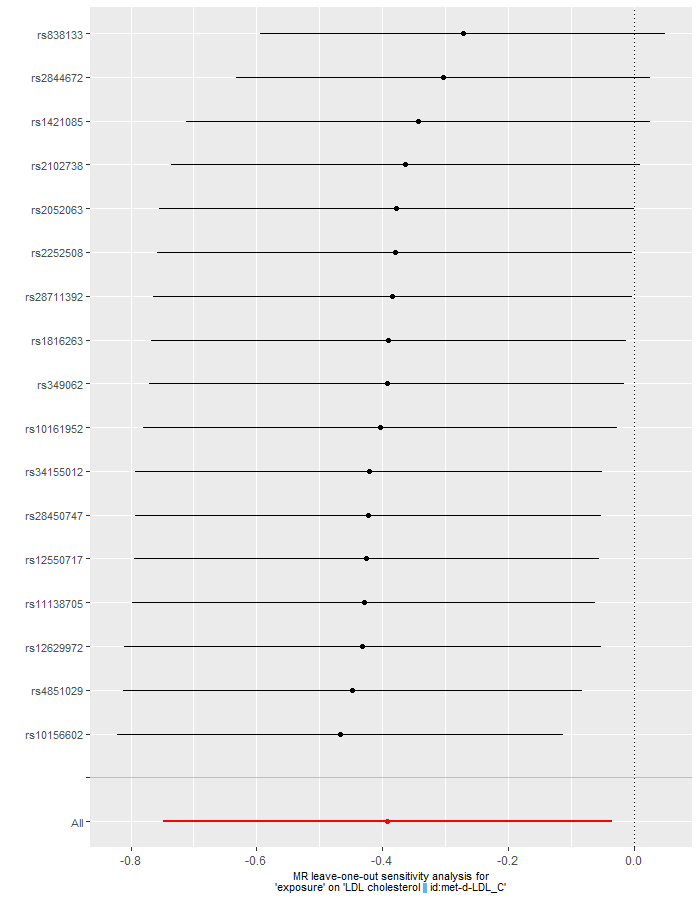


**(E)Total triglycerides**


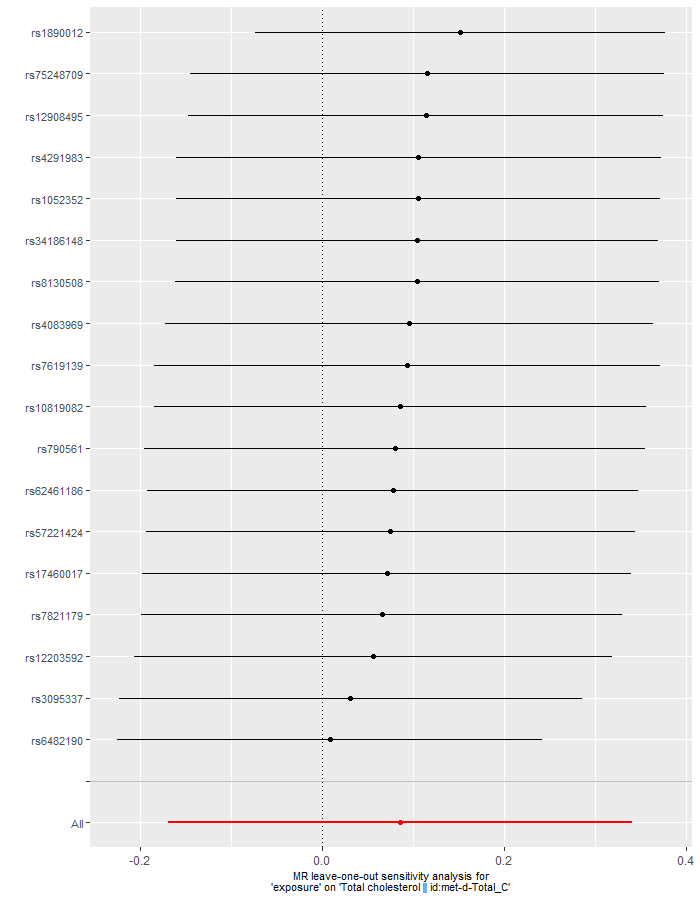


**(F)Total cholesterol**


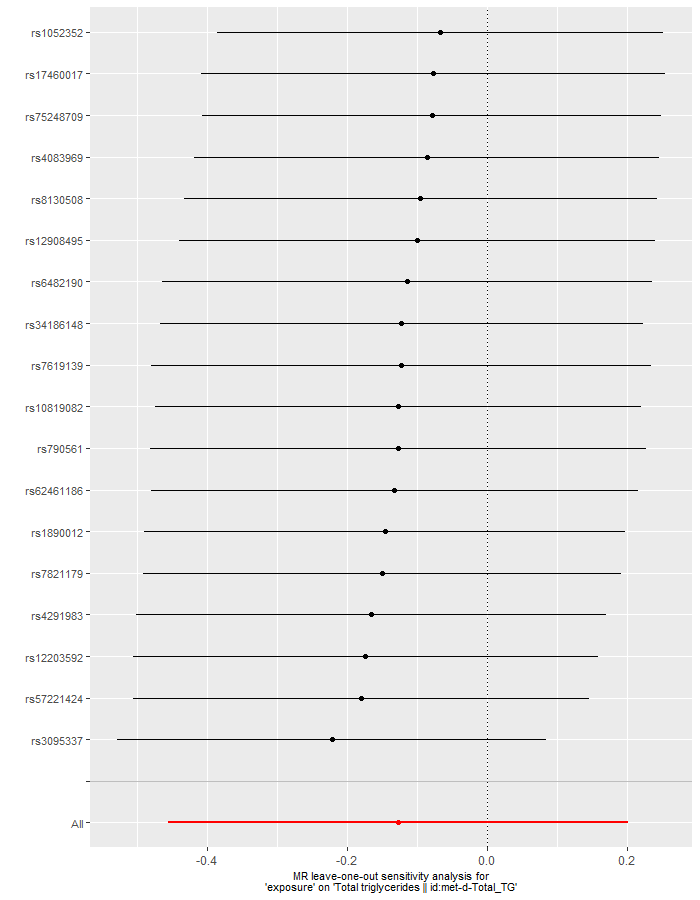


**Figure S13. Estimates given as odds ratios (ORs) and 95% confidence intervals for the effect of raw vegetable intake (IV from UK biobank and MRC-IEU) on ischemic cardio-cerebral vascular diseases**


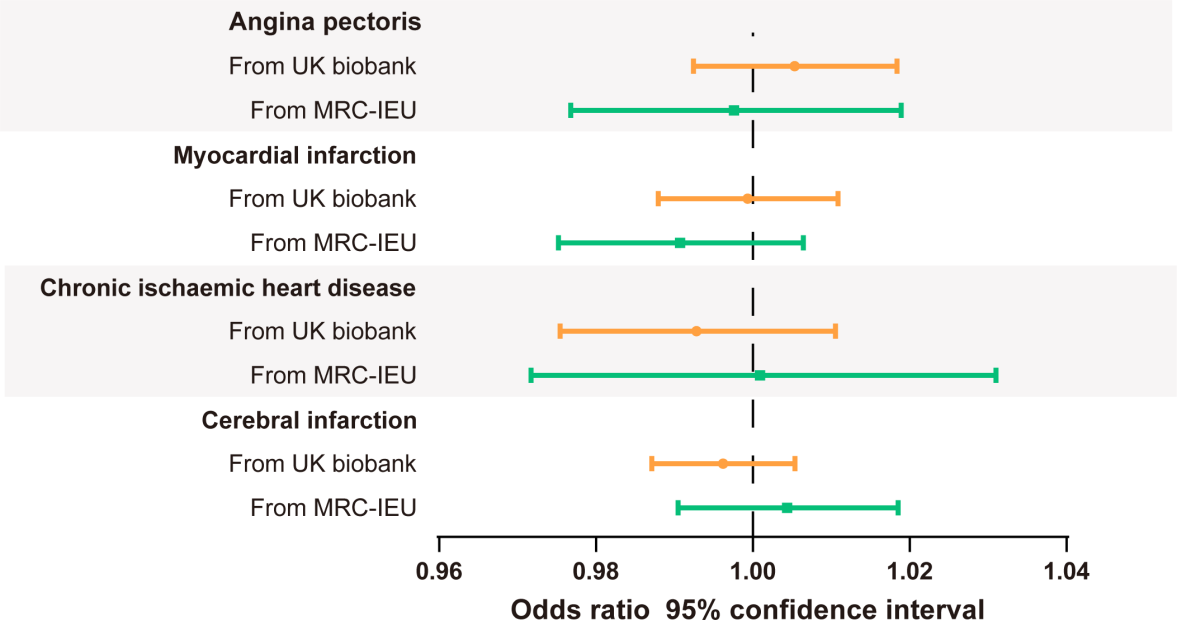


**Figure S14. Estimates given as odds ratios (ORs) and 95% confidence intervals for the effect of cooked vegetable intake (IV from UK biobank and MRC-IEU) on ischemic cardio-cerebral vascular diseases**


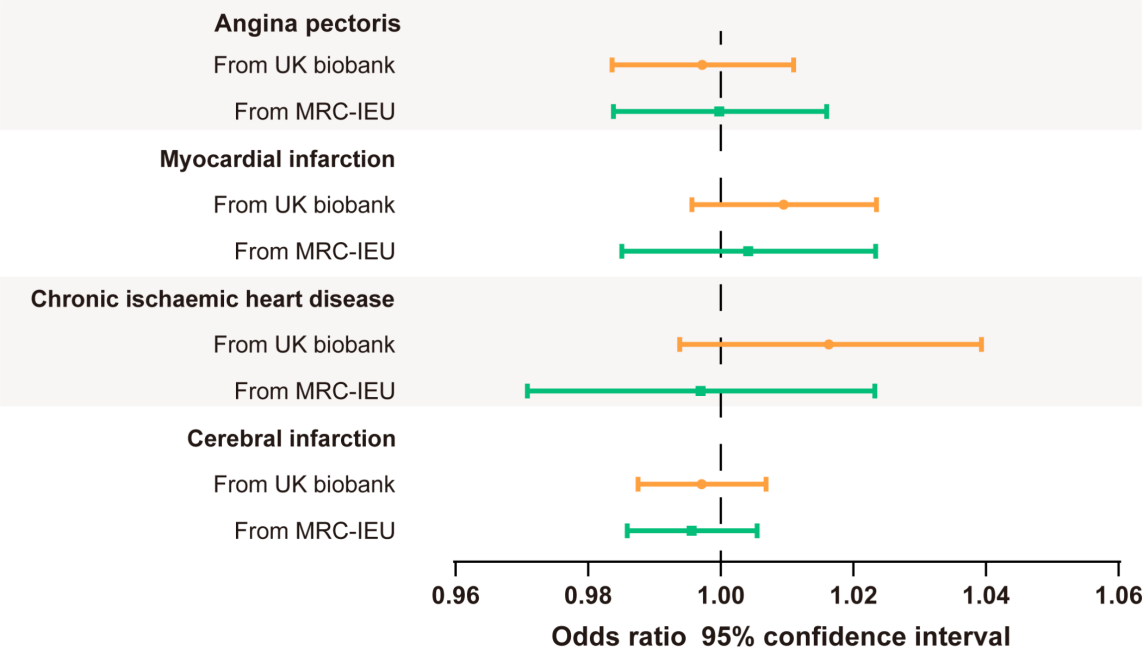


**Figure S15. Estimates given as beta and 95% confidence intervals for the effect of raw vegetable intake (IV from UK biobank and MRC-IEU) on lipid profiles**


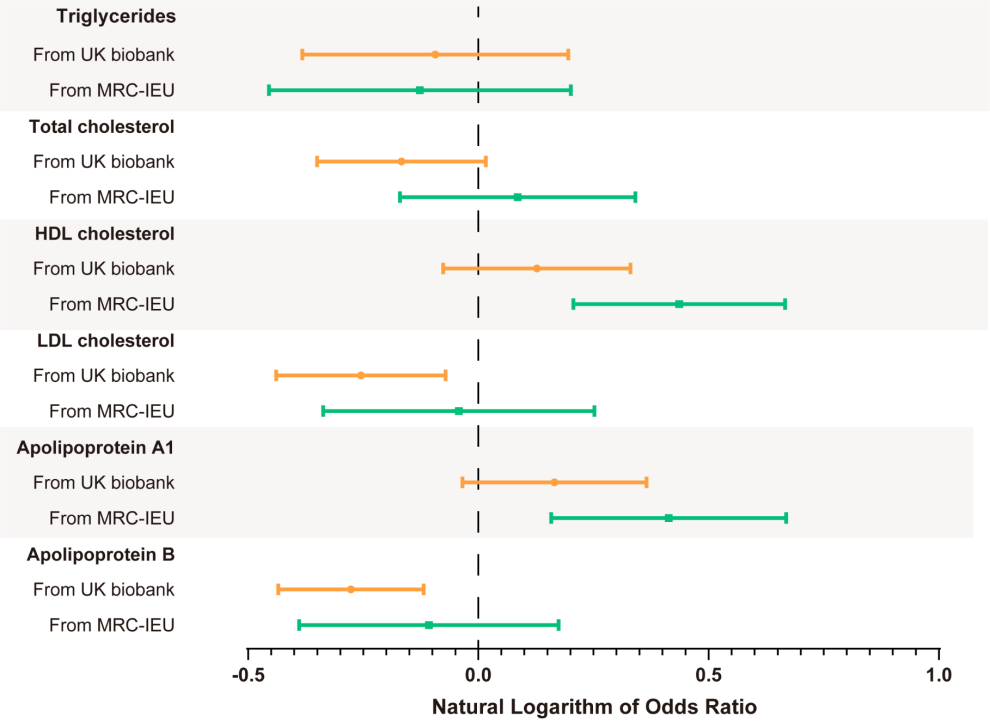


**Figure S16. Estimates given as beta and 95% confidence intervals for the effect of cooked vegetable intake (IV from UK biobank and MRC-IEU) on lipid profiles**


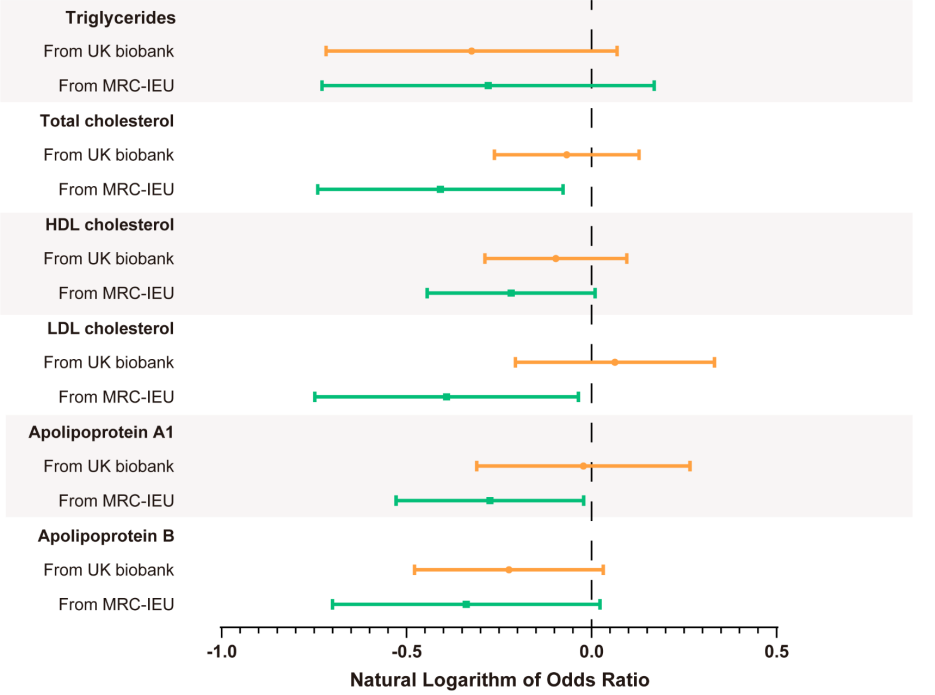

Supplement: Supplementary file 1 [file Data_Sheet_1.zip › Supplementary Figure.docx]
